# Supplementary material for: Ribonucleotide incorporation in yeast genomic DNA shows preference for cytosine and guanosine preceded by deoxyadenosine
Source: Nat Commun. 2020 May 15;11:2447. doi: 10.1038/s41467-020-16152-5 (PMC7229183; doi:10.1038/s41467-020-16152-5)
Supplement: Supplementary file 1 — Supplementary Information [file 41467_2020_16152_MOESM1_ESM.pdf]

Supplementary Information for

Ribonucleotide incorporation in yeast genomic DNA shows preference for cytosine and guanosine preceded by deoxyadenosine

Balachander, Gombolay, Yang, Xu et al.

Supplementary Table 1. Yeast strains used in this study.

Strain	Relevant genotype	Source
E134 (KK-44)	*MATα ade5-1 lys2-14A trp1-289 his7-2 leu2-3,112 ura3-52*	Koh *et al.*, 2015([1])
KK-100	KK-44 *rnh201*Δ::*hygMX4*	Koh *et al.*, 2015([1])
KK-172	KK-44 *rnh1*Δ::*kanMX4*	this study
SB-286	KK-44 *rnh202-FF346,347AA*	this study
BY4742 (KK-2)	*MATα his3*Δ*1 leu2*Δ*0 lys2*Δ*0 ura3* Δ0	Storici *et al.*, 2001([2])
SB-305	KK-2 *rnh201*Δ::*hygMX4*	this study
SB-285	KK-2 *rnh202-FF346,347AA*	this study
SB-311	KK-2 *rnh201-P45D Y219A*	this study
BY4741 (SB-292)	*MATa his3-1 leu2-0 met15-0 ura3-0*	Brachmann *et al*., 1998([3])
YFP17 (SB-288)	*ho*Δ *hml*Δ::*ADE1 mata*Δ::*hisG Δhmr*::*ADE1 ade1 leu2-3,1122 lys5 trp1*::*hisG ura3-52 ade3*::*GAL*::*HO*	Keskin *et al.*, 2014([4])
SB-293	SB-293 *rnh201*Δ::*hygMX4*	this study
W303 (SB-316)	*MATa can1-100 his3-11,15 leu2-3,112 trp1-1 ura3-1 ade2-1*	Ralser *et al.*, 2012([5])
S288C (SB-313)	*MATα SUC2 gal2 mal2 mel flo8-1 hap1 ho bio1 bio6*	Mortimer and Johnston (1986)([6])
DG2204 (HK-692)	*MATa LYS+ trp1 ADE+ LEU+ ura3 his3-Δ200 hisG Gal+ Spo+ Ty-less* (*S. paradoxus*)	Garfinkel *et* al., 2005([7])
HK-705	HK-692 *rnh201*Δ::*hygMX4* (*S. paradoxus*)	this study
JZ105 (KK-154)	*Mat1M mat2,3*Δ::*LEU2 ade6-210 leu1-32 ura4-D18 his2* (*S. pombe*)	Vengrova *et al.*, 2004([8])
HK-983	KK-154 *rnh201*Δ::*kanMX4* (*S. pombe*)	this study

Yeast strains used in this study for ribose-seq library construction and their corresponding genotype. The strains include *S. cerevisiae* strains, as well as strains of *S. paradoxus* and *S. pombe,* as indicated in parenthesis.

Supplementary Table 2. Oligonucleotides used in this study.

Name	Size	Sequence
202PIP.F	86	5’AAAACCAAAAGTAGCCATAGGAAAAGGGGCCATTGATGGA**GCTGCT**AAACGTAAGTAGCTAGTATCATAATTAAACAGCAATTTGA
202PIP.R	86	5’TCAAATTGCTGTTTAATTATGATACTAGCTACTTACGTTT**AGCAGC**TCCATCAATGGCCCCTTTTCCTATGGCTACTTTTGGTTTT
RNH P45D.60	60	5’AATGGGTATCGATGAAGCTGGCAGAGGG**GA**CGTATTAGGGCCAATGGTCTACGCAGTAG
RNH Y219A.60	60	5’GGGATCCCGATGAGATCCTGGGTTCTGGA**GC**CCCCTCCGACCCGAAGACAGTCGCATGGC
Adaptor.L1 *	65	5’ P-NNC CGN NNN NNA GAT CGG AAG AGC GTC GTG TAG GGA AAG AGT GTT GAT AGA TCC GTG TCG CAA CT
Adaptor.L2 *	65	5’ P-NNT GAN NNN NNA GAT CGG AAG AGC GTC GTG TAG GGA AAG AGT GTT GAT AGA TCC GTG TCG CAA CT
Adaptor.S *	25	5’ P-GTT GCG ACA CGG ATC TAT CAA CAC T -Am
PCR.1	54	5’ GTG ACT GGA GTT CAG ACG TGT GCT CTT CCG ATC TTG ATA GAT CCG TGT CGC AAC
PCR.2	20	5’ ACA CTC TTT CCC TAC ACG AC
PCR.701	53	5’ CAA GCA GAA GAC GGC ATA CGA GAT **CGA GTA AT**G TGA CTG GAG TTC AGA CGT GT
PCR.702	53	5’ CAA GCA GAA GAC GGC ATA CGA GAT **TCT CCG GA**G TGA CTG GAG TTC AGA CGT GT
PCR.501	57	5’ AAT GAT ACG GCG ACC GAG ATC TAC AC**T ATA GCC T**AC ACT CTT TCC CTA CAC GAC
PCR.502	57	5’ AAT GAT ACG GCG ACC GAG ATC TAC AC**A TAG AGG C**AC ACT CTT TCC CTA CAC GAC
PCR.503	57	5’ AAT GAT ACG GCG ACC GAG ATC TAC AC**C CTA TCC T**AC ACT CTT TCC CTA CAC GAC
PCR.504	57	5’ AAT GAT ACG GCG ACC GAG ATC TAC AC**G GCT CTG A**AC ACT CTT TCC CTA CAC GAC

PCR.505	57	5' AAT GAT ACG GCG ACC GAG ATC TAC AC**A GGC GAA G**AC ACT CTT TCC CTA CAC GAC
PCR.506	57	5' AAT GAT ACG GCG ACC GAG ATC TAC AC**T AAT CTT A**AC ACT CTT TCC CTA CAC GAC
PCR.507	57	5' AAT GAT ACG GCG ACC GAG ATC TAC AC**C AGG ACG T**AC ACT CTT TCC CTA CAC GAC
PCR.508	57	5' AAT GAT ACG GCG ACC GAG ATC TAC AC**G TAC TGA C**AC ACT CTT TCC CTA CAC GAC

Name, length, and sequence of oligonucleotides used in this study are presented. The bold and underlined letters in oligonucleotides 202.PIP.F, 202.PIP.R, RNH P45D.60, and RNH Y219A.60 indicate the specific mutations that were introduced in *RNH202* or *RNH201* genes. All bold letters in the PCR primers indicate the specific sequence of index used in sequencing. P and Am indicate end modifications of phosphate and amino groups, respectively. All oligonucleotides were desalted, except those marked with an asterisks (*), which were HPLC purified. 202.PIP.F, 202.PIP.R, RNH P45D.60, and RNH Y219A.60 were synthesized by Eurofins Technologies, and all other oligonucleotides were synthesized by IDT.

Supplementary Table 3. Concentration of dNTPs and rNTPs in yeast cells.

Sample	Strain	Normalized concentration of dNTPs and rNTPS (pmols per 10^7 cells)							
S. cerevisiae		dATP	dCTP	dGTP	dTTP	rATP	rCTP	rGTP	rUTP
1	E134	1.1	BLOQ	0.8	1.5	139	51.4	55.5	70.6
2	E134	0.7	0.45	0.59	0.94	78.7	28.4	34.3	45
3	E134	1.51	0.41	2.28	1.89	213.6	126.7	159.4	141.6
4	BY4742	0.082	BLOQ	0.073	0.14	26.8	6.94	9.65	9.99
5	BY4742	0.16	0.063	0.13	0.2	40.3	9.2	14.4	14.4
6	BY4742	0.15	0.1	0.14	0.24	45.7	11.3	17.6	16.4
S. pombe									
7	JZ105	0.046	0.02	0.11	0.058	38.7	4.65	19.3	9.13
8	JZ105	0.063	0.015	0.14	0.073	47.1	5.4	24.7	11
9	JZ105	0.064	BLOQ	0.12	0.063	45.5	5.13	22.4	10.5

The levels of dNTPs and rNTPs extracted from known numbers of yeast cells of *S. cerevisiae* strain E134 and BY4742 and *S. pombe* strain JZ105 were determined by LC-MS/MS methods (see Methods). BLOQ: Below limit of quantification. The cellular level of each nucleotide was normalized for 10^7 cells.

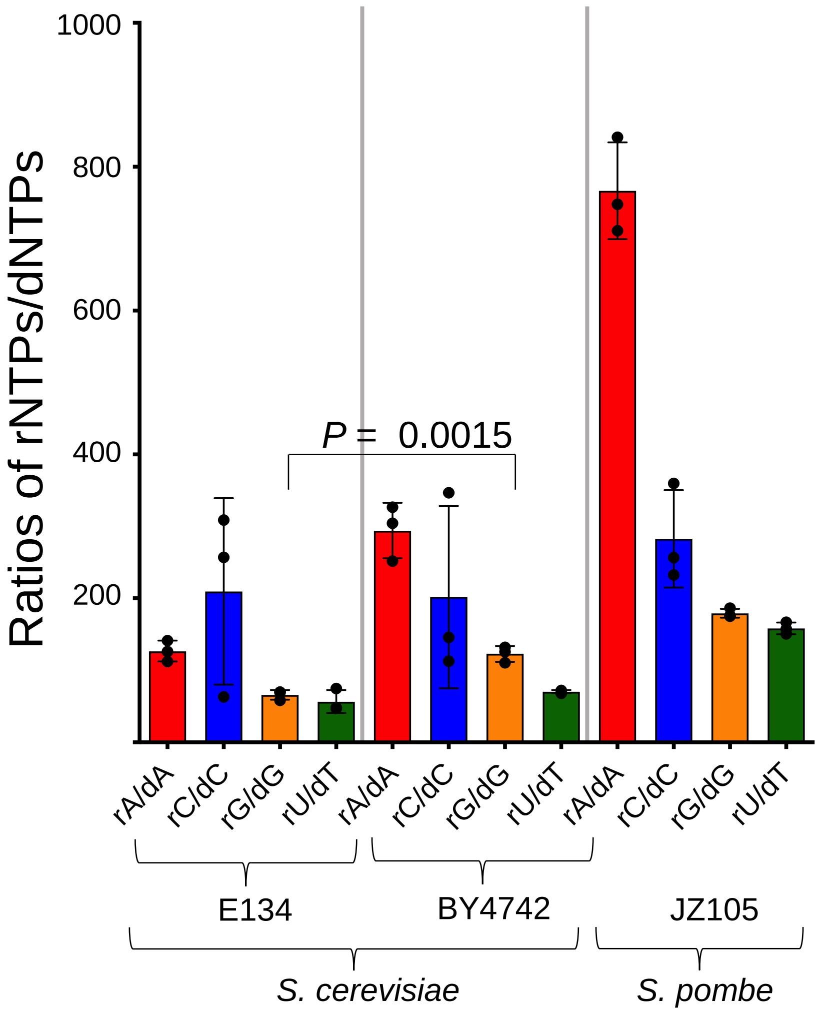

Supplementary Figure 1. Measurements of dNTP and rNTP levels in yeast cells. The dNTP and rNTP levels determined in Supplementary Table 3 were used to calculate the rNTP/dNTP ratios. Shown are mean and standard deviation of three independent measures for each sample. Ratios for dCTP levels below the limit of quantification were calculated by using the limit of quantification for dCTP (0.02 pmole) relative to the number of cells used (10^6 for sample 1 and 10^7 for samples 4 and 9) in the LC-MS/MS method. Ratios of rGTP/dGTP for E134 and BY4742 were compared with each other using the two-tailed t-Test; the corresponding *P* value is shown.

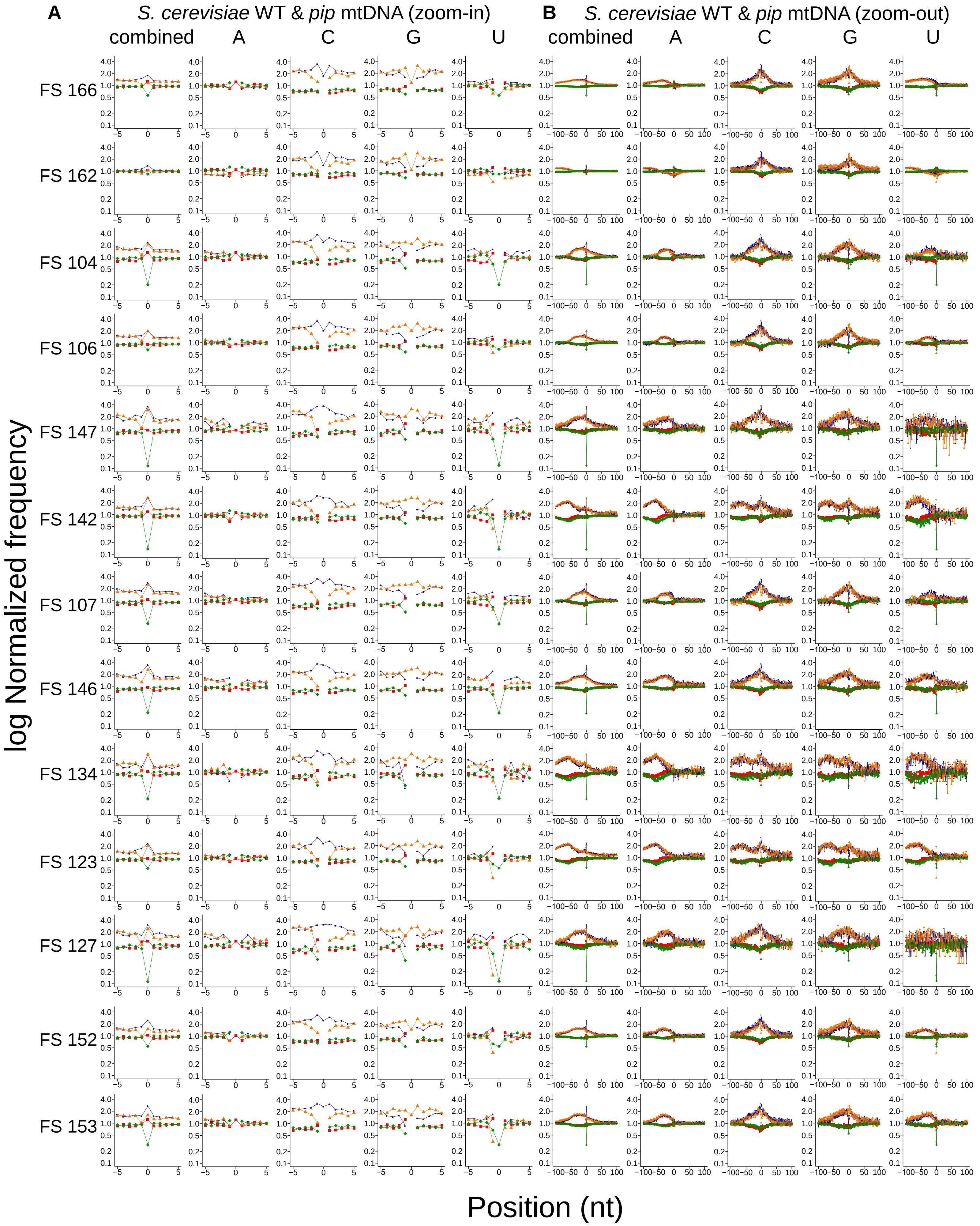

A
S. cerevisiae WT & pip mtDNA (zoom-in)
B
S. cerevisiae WT & pip mtDNA (zoom-out)
combined
A
C
G
U
FS 166
FS 162
FS 104
FS 106
FS 147
FS 142
FS 107
FS 146
FS 134
FS 123
FS 127
FS 152
FS 153
log Normalized frequency
Position (nt)

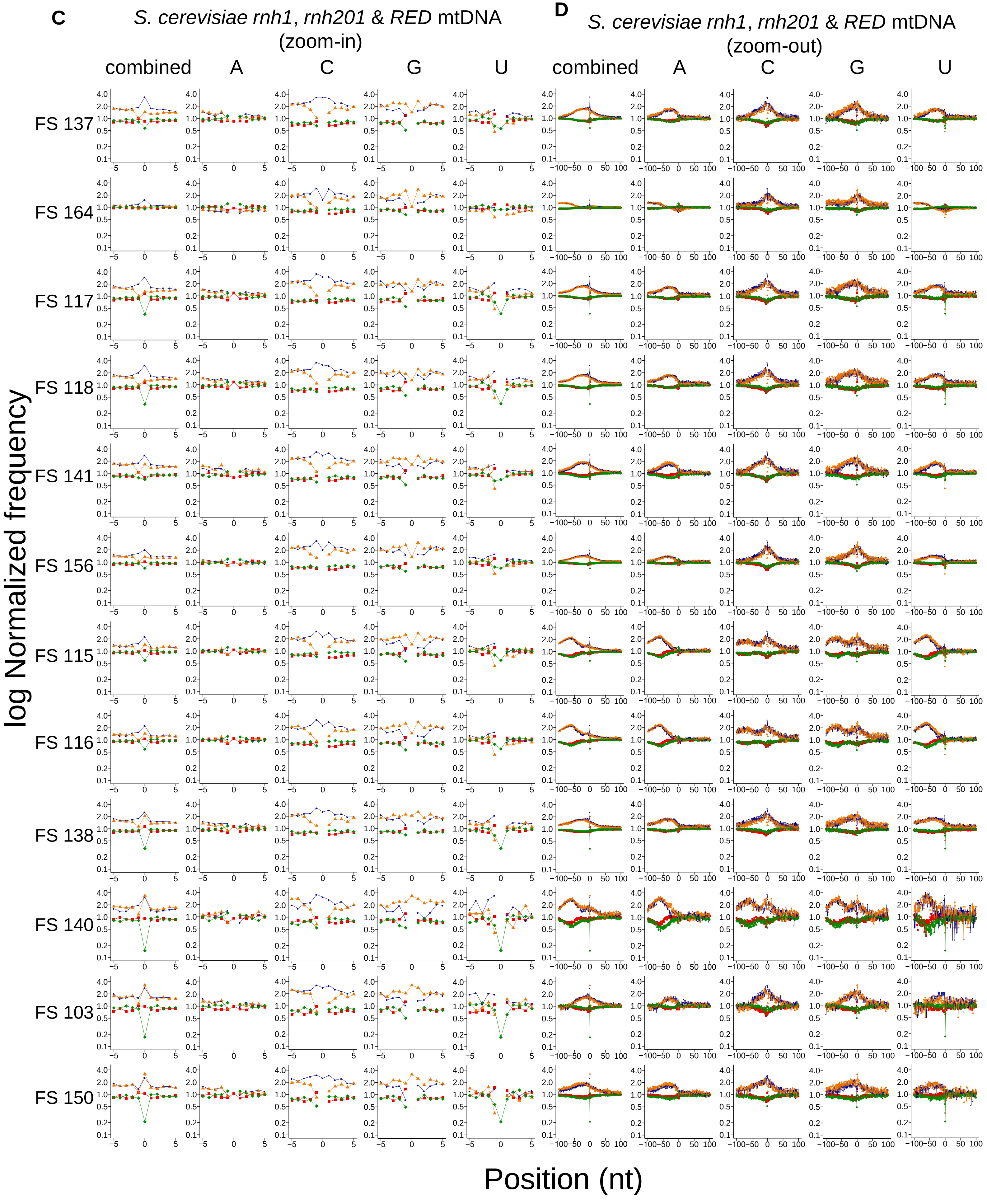
C
S. cerevisiae rnh1, rnh201 & RED mtDNA (zoom-in)
D
S. cerevisiae rnh1, rnh201 & RED mtDNA (zoom-out)
combined
A
C
G
U
FS 137
FS 164
FS 117
FS 118
FS 141
FS 156
FS 115
FS 116
FS 138
FS 140
FS 103
FS 150
log Normalized frequency
Position (nt)

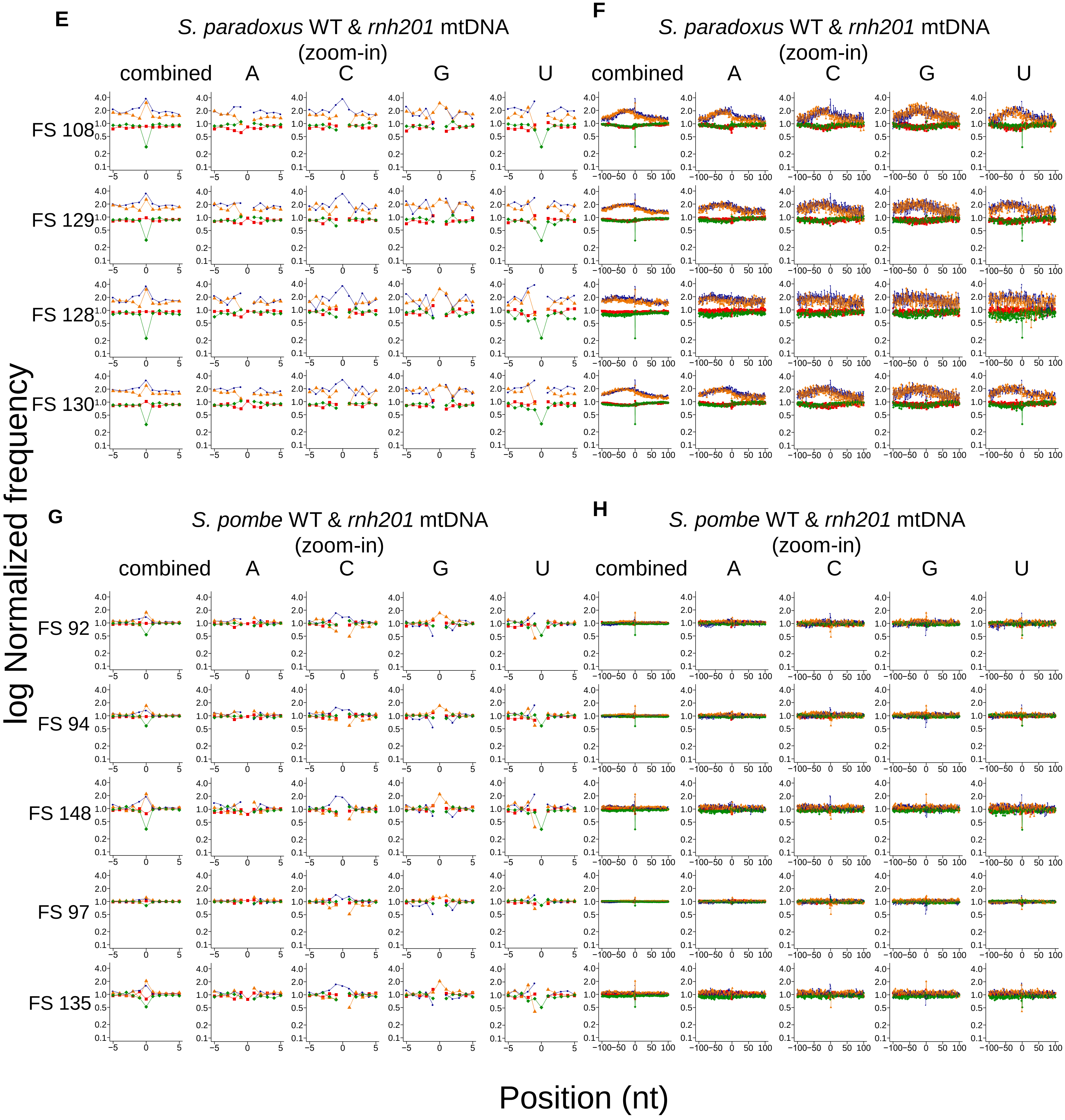
E
S. paradoxus WT & rnh201 mtDNA (zoom-in)
F
S. paradoxus WT & rnh201 mtDNA (zoom-in)
combined
A
C
G
U
FS 108
FS 129
FS 128
FS 130
G
S. pombe WT & rnh201 mtDNA (zoom-in)
H
S. pombe WT & rnh201 mtDNA (zoom-in)
FS 92
FS 94
FS 148
FS 97
FS 135
log Normalized frequency
Position (nt)

Supplementary Figure 2. Nucleotide plots of all mitochondrial libraries. (**A-H**) Plots of normalized nucleotide frequencies relative to mapped positions of sequences from the (**A,B**) 11 wild-type and 2 *pip S. cerevisiae* mitochondrial libraries generated in this study, combined and single, (**A**) zoom-in, and (**B**) zoomed-out plots; (**C,D**) 2 *rnh1*, 8 *rnh201* and 2 *RED S. cerevisiae* mitochondrial libraries combined and single, (**C**) zoom-in, and (**D**) zoomed-out plots; (**E,F**) 3 *S. paradoxus* wild-type and 1 *rnh201* mitochondrial libraries combined and single, (**E**) zoom-in, and (**F**) zoomed-out plots; (**G,H**) 3 *S. pombe* wild-type and 2 *rnh201* mitochondrial libraries combined and single, (**G**) zoom-in, and (**H**) zoomed-out plots. Position 0 on the x-axis represents the site of rNMP incorporation, - and + positions represent upstream and downstream dNMPs, respectively. The y-axis shows the frequency of each type of nucleotide present in the ribose-seq data normalized to the frequency of the corresponding nucleotide present in the reference genome of the indicted yeast species. Red square, A; blue circle, C; orange triangle, G; and green rhombus, U.

A

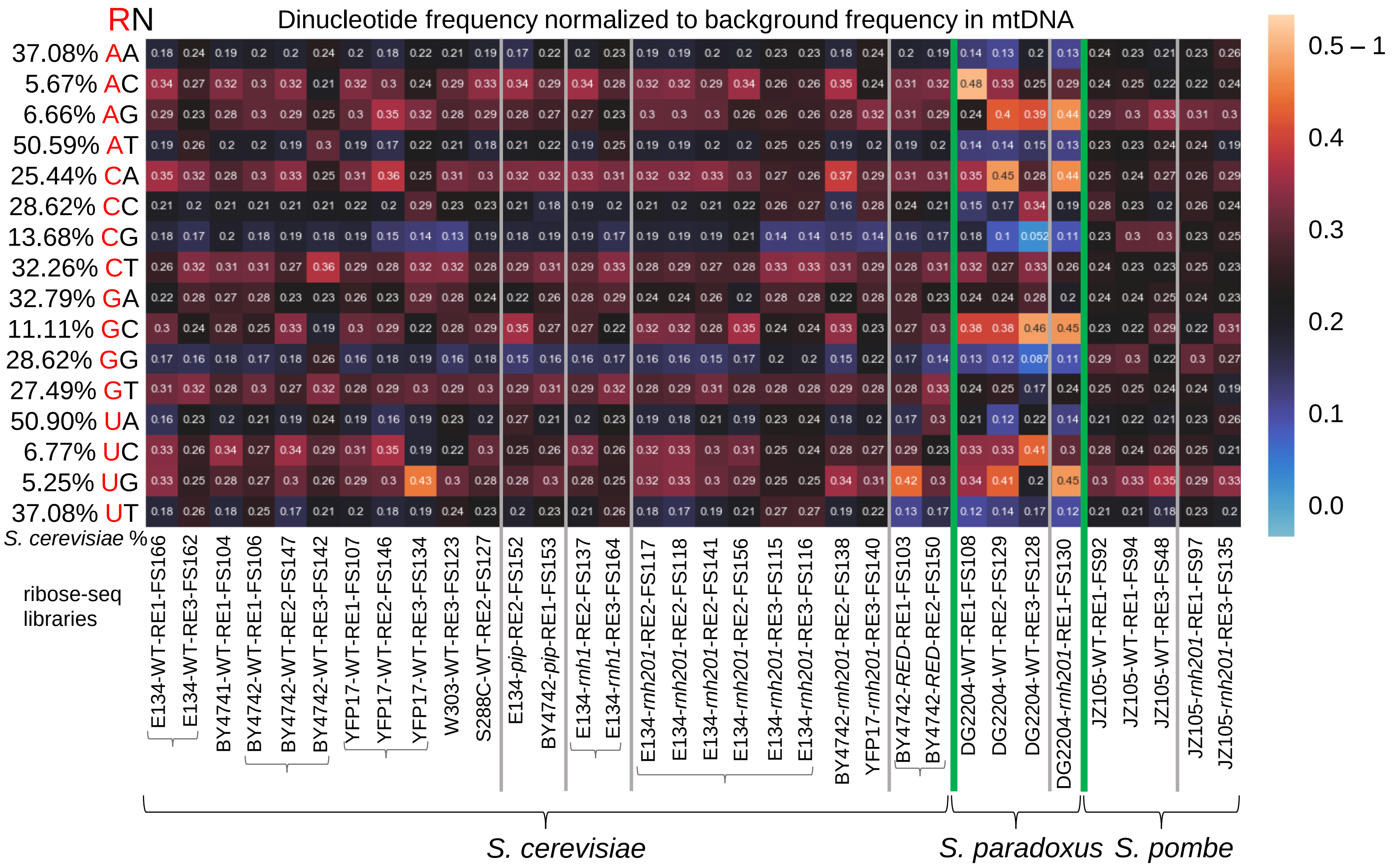

RN
Dinucleotide frequency normalized to background frequency in mtDNA
37.08% AA
5.67% AC
6.66% AG
50.59% AT
25.44% CA
28.62% CC
13.68% CG
32.26% CT
32.79% GA
11.11% GC
28.62% GG
27.49% GT
50.90% UA
6.77% UC
5.25% UG
37.08% UT
S. cerevisiae %
ribose-seq libraries
E134-WT-RE1-FS166
E134-WT-RE3-FS162
BY4741-WT-RE1-FS104
BY4742-WT-RE1-FS106
BY4742-WT-RE2-FS147
BY4742-WT-RE3-FS142
YFP17-WT-RE1-FS107
YFP17-WT-RE2-FS146
YFP17-WT-RE3-FS134
W303-WT-RE3-FS123
S288C-WT-RE2-FS127
E134-pip-RE2-FS152
BY4742-pip-RE1-FS153
E134-rnh1-RE2-FS137
E134-rnh1-RE3-FS164
E134-rnh201-RE2-FS117
E134-rnh201-RE2-FS118
E134-rnh201-RE2-FS141
E134-rnh201-RE2-FS156
E134-rnh201-RE3-FS115
E134-rnh201-RE3-FS116
BY4742-rnh201-RE2-FS138
YFP17-rnh201-RE3-FS140
BY4742-RED-RE1-FS103
BY4742-RED-RE2-FS150
DG2204-WT-RE1-FS108
DG2204-WT-RE2-FS129
DG2204-WT-RE3-FS128
DG2204-rnh201-RE1-FS130
JZ105-WT-RE1-FS92
JZ105-WT-RE1-FS94
JZ105-WT-RE3-FS48
JZ105-rnh201-RE1-FS97
JZ105-rnh201-RE3-FS135
0.5 – 1
0.4
0.3
0.2
0.1
0.0
S. cerevisiae
S. paradoxus
S. pombe

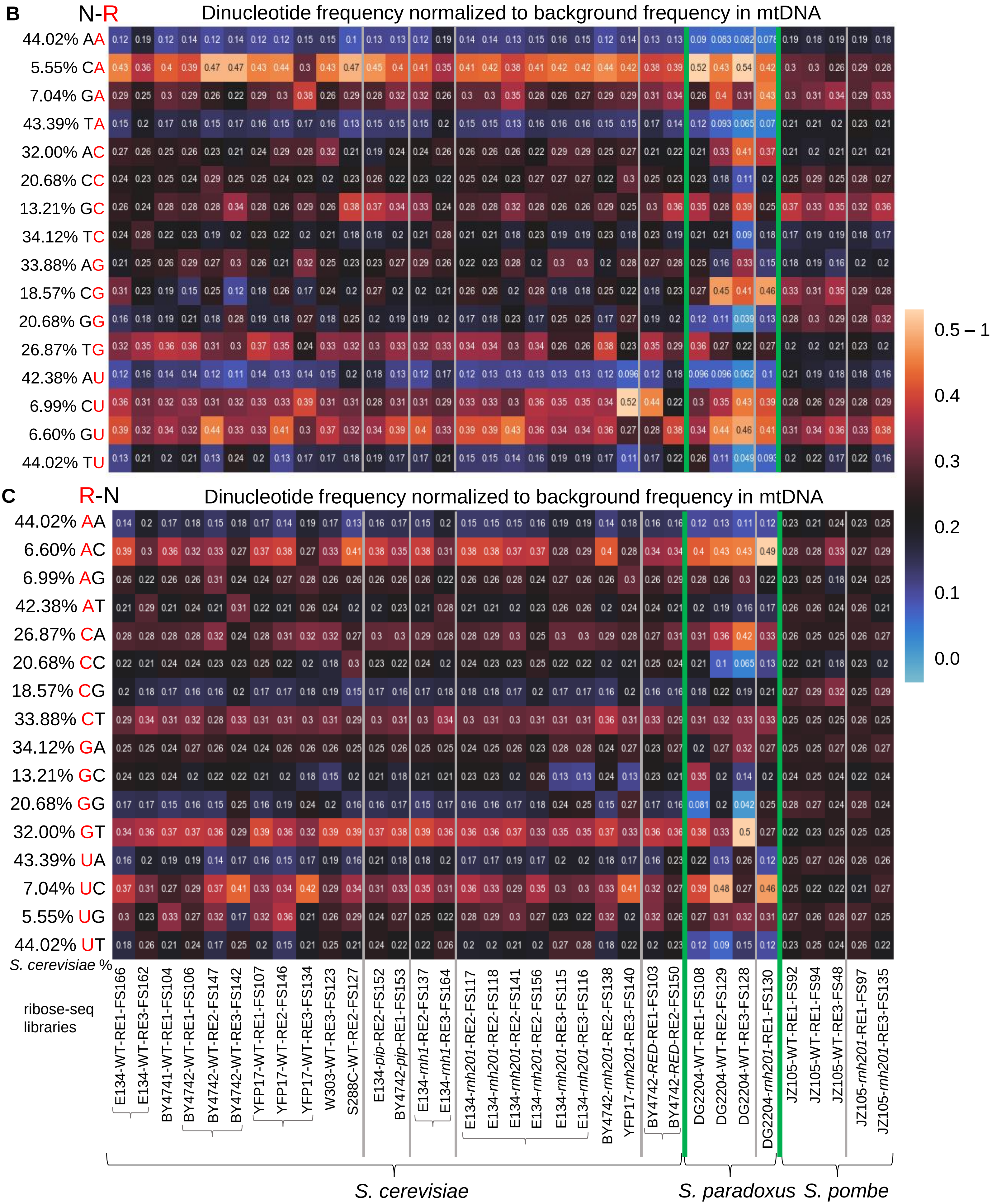
B
N-R
Dinucleotide frequency normalized to background frequency in mtDNA
44.02% AA
5.55% CA
7.04% GA
43.39% TA
32.00% AC
20.68% CC
13.21% GC
34.12% TC
33.88% AG
18.57% CG
20.68% GG
26.87% TG
42.38% AU
6.99% CU
6.60% GU
44.02% TU
C
R-N
Dinucleotide frequency normalized to background frequency in mtDNA
44.02% AA
6.60% AC
6.99% AG
42.38% AT
26.87% CA
20.68% CC
18.57% CG
33.88% CT
34.12% GA
13.21% GC
20.68% GG
32.00% GT
43.39% UA
7.04% UC
5.55% UG
44.02% UT
S. cerevisiae %
ribose-seq libraries
E134-WT-RE1-FS166
E134-WT-RE3-FS162
BY4741-WT-RE1-FS104
BY4742-WT-RE1-FS106
BY4742-WT-RE2-FS147
BY4742-WT-RE3-FS142
YFP17-WT-RE1-FS107
YFP17-WT-RE2-FS146
YFP17-WT-RE3-FS134
W303-WT-RE3-FS123
S288C-WT-RE2-FS127
E134-pip-RE2-FS152
BY4742-pip-RE1-FS153
E134-rnh1-RE2-FS137
E134-rnh1-RE3-FS164
E134-rnh201-RE2-FS117
E134-rnh201-RE2-FS118
E134-rnh201-RE2-FS141
E134-rnh201-RE2-FS156
E134-rnh201-RE3-FS115
E134-rnh201-RE3-FS116
BY4742-rnh201-RE2-FS138
YFP17-rnh201-RE3-FS140
BY4742-RED-RE1-FS103
BY4742-RED-RE2-FS150
DG2204-WT-RE1-FS108
DG2204-WT-RE2-FS129
DG2204-WT-RE3-FS128
DG2204-rnh201-RE1-FS130
JZ105-WT-RE1-FS92
JZ105-WT-RE1-FS94
JZ105-WT-RE3-FS48
JZ105-rnh201-RE1-FS97
JZ105-rnh201-RE3-FS135
S. cerevisiae
S. paradoxus
S. pombe
0.5 – 1
0.4
0.3
0.2
0.1
0.0

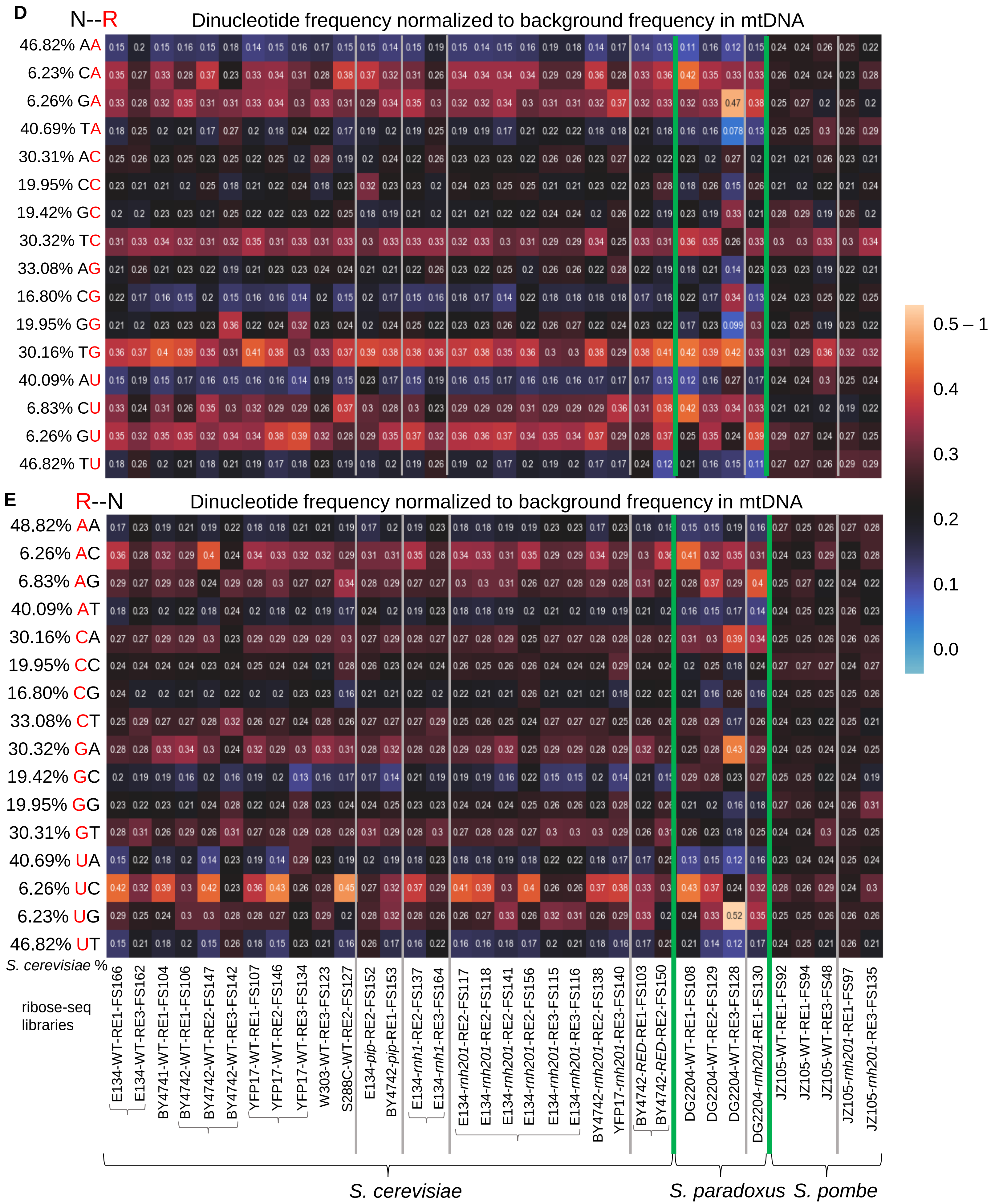
D
N--R
Dinucleotide frequency normalized to background frequency in mtDNA
46.82% AA
6.23% CA
6.26% GA
40.69% TA
30.31% AC
19.95% CC
19.42% GC
30.32% TC
33.08% AG
16.80% CG
19.95% GG
30.16% TG
40.09% AU
6.83% CU
6.26% GU
46.82% TU
E
R--N
Dinucleotide frequency normalized to background frequency in mtDNA
48.82% AA
6.26% AC
6.83% AG
40.09% AT
30.16% CA
19.95% CC
16.80% CG
33.08% CT
30.32% GA
19.42% GC
19.95% GG
30.31% GT
40.69% UA
6.26% UC
6.23% UG
46.82% UT
S. cerevisiae %
ribose-seq libraries
E134-WT-RE1-FS166
E134-WT-RE3-FS162
BY4741-WT-RE1-FS104
BY4742-WT-RE1-FS106
BY4742-WT-RE2-FS147
BY4742-WT-RE3-FS142
YFP17-WT-RE1-FS107
YFP17-WT-RE2-FS146
YFP17-WT-RE3-FS134
W303-WT-RE3-FS123
S288C-WT-RE2-FS127
E134-pip-RE2-FS152
BY4742-pip-RE1-FS153
E134-rnh1-RE2-FS137
E134-rnh1-RE3-FS164
E134-rnh201-RE2-FS117
E134-rnh201-RE2-FS118
E134-rnh201-RE2-FS141
E134-rnh201-RE2-FS156
E134-rnh201-RE3-FS115
E134-rnh201-RE3-FS116
BY4742-rnh201-RE2-FS138
YFP17-rnh201-RE3-FS140
BY4742-RED-RE1-FS103
BY4742-RED-RE2-FS150
DG2204-WT-RE1-FS108
DG2204-WT-RE2-FS129
DG2204-WT-RE3-FS128
DG2204-rnh201-RE1-FS130
JZ105-WT-RE1-FS92
JZ105-WT-RE1-FS94
JZ105-WT-RE3-FS48
JZ105-rnh201-RE1-FS97
JZ105-rnh201-RE3-FS135
S. cerevisiae
S. paradoxus
S. pombe
0.5 – 1
0.4
0.3
0.2
0.1
0.0

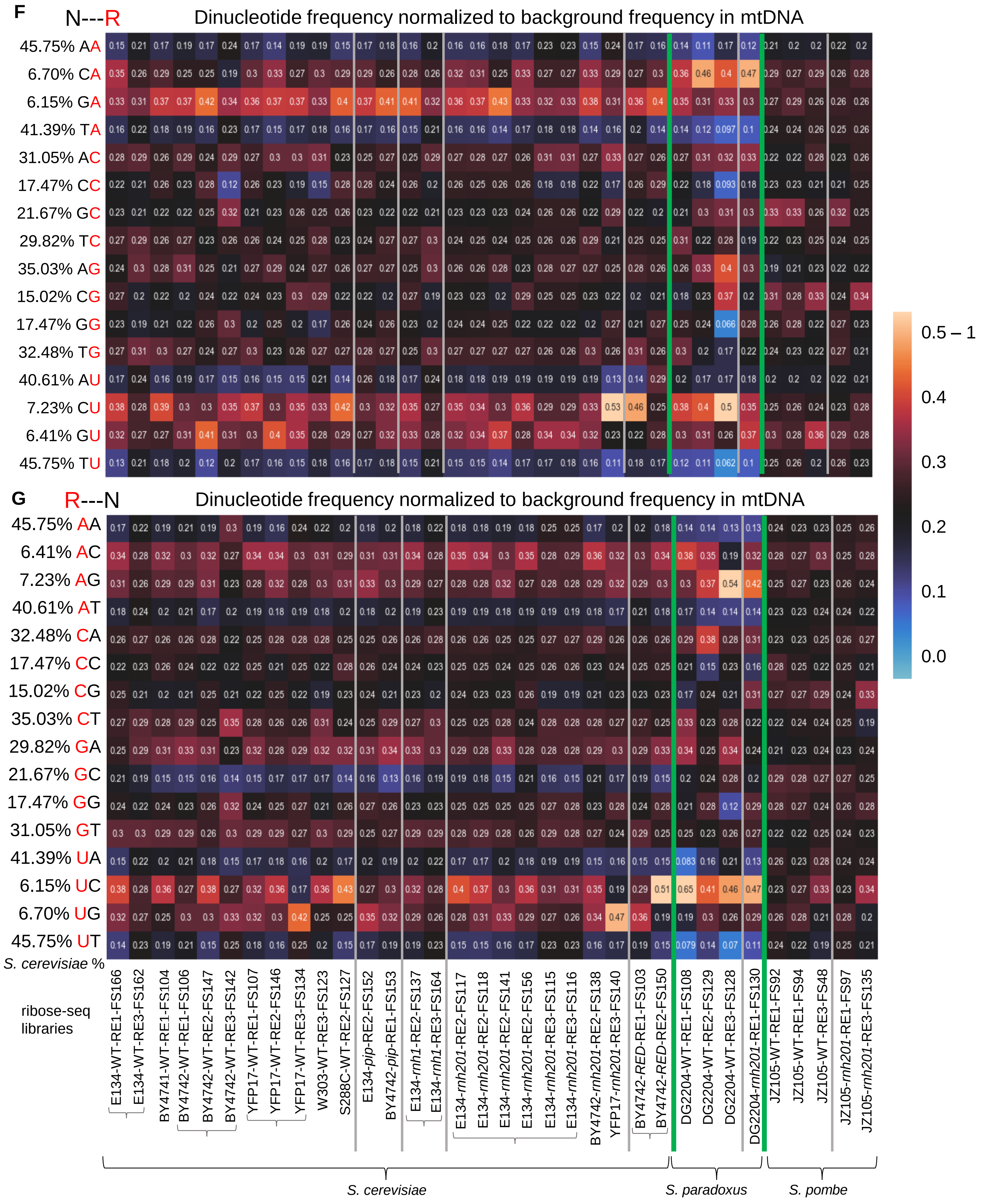
F
N---R
Dinucleotide frequency normalized to background frequency in mtDNA
45.75% AA
6.70% CA
6.15% GA
41.39% TA
31.05% AC
17.47% CC
21.67% GC
29.82% TC
35.03% AG
15.02% CG
17.47% GG
32.48% TG
40.61% AU
7.23% CU
6.41% GU
45.75% TU
G
R---N
Dinucleotide frequency normalized to background frequency in mtDNA
45.75% AA
6.41% AC
7.23% AG
40.61% AT
32.48% CA
17.47% CC
15.02% CG
35.03% CT
29.82% GA
21.67% GC
17.47% GG
31.05% GT
41.39% UA
6.15% UC
6.70% UG
45.75% UT
S. cerevisiae %
ribose-seq libraries
0.5 – 1
0.4
0.3
0.2
0.1
0.0
E134-WT-RE1-FS166
E134-WT-RE3-FS162
BY4741-WT-RE1-FS104
BY4742-WT-RE1-FS106
BY4742-WT-RE2-FS147
BY4742-WT-RE3-FS142
YFP17-WT-RE1-FS107
YFP17-WT-RE2-FS146
YFP17-WT-RE3-FS134
W303-WT-RE3-FS123
S288C-WT-RE2-FS127
E134-pip-RE2-FS152
BY4742-pip-RE1-FS153
E134-rnh1-RE2-FS137
E134-rnh1-RE3-FS164
E134-rnh201-RE2-FS117
E134-rnh201-RE2-FS118
E134-rnh201-RE2-FS141
E134-rnh201-RE2-FS156
E134-rnh201-RE3-FS115
E134-rnh201-RE3-FS116
BY4742-rnh201-RE2-FS138
YFP17-rnh201-RE3-FS140
BY4742-RED-RE1-FS103
BY4742-RED-RE2-FS150
DG2204-WT-RE1-FS108
DG2204-WT-RE2-FS129
DG2204-WT-RE3-FS128
DG2204-rnh201-RE1-FS130
JZ105-WT-RE1-FS92
JZ105-WT-RE1-FS94
JZ105-WT-RE3-FS48
JZ105-rnh201-RE1-FS97
JZ105-rnh201-RE3-FS135
S. cerevisiae
S. paradoxus
S. pombe

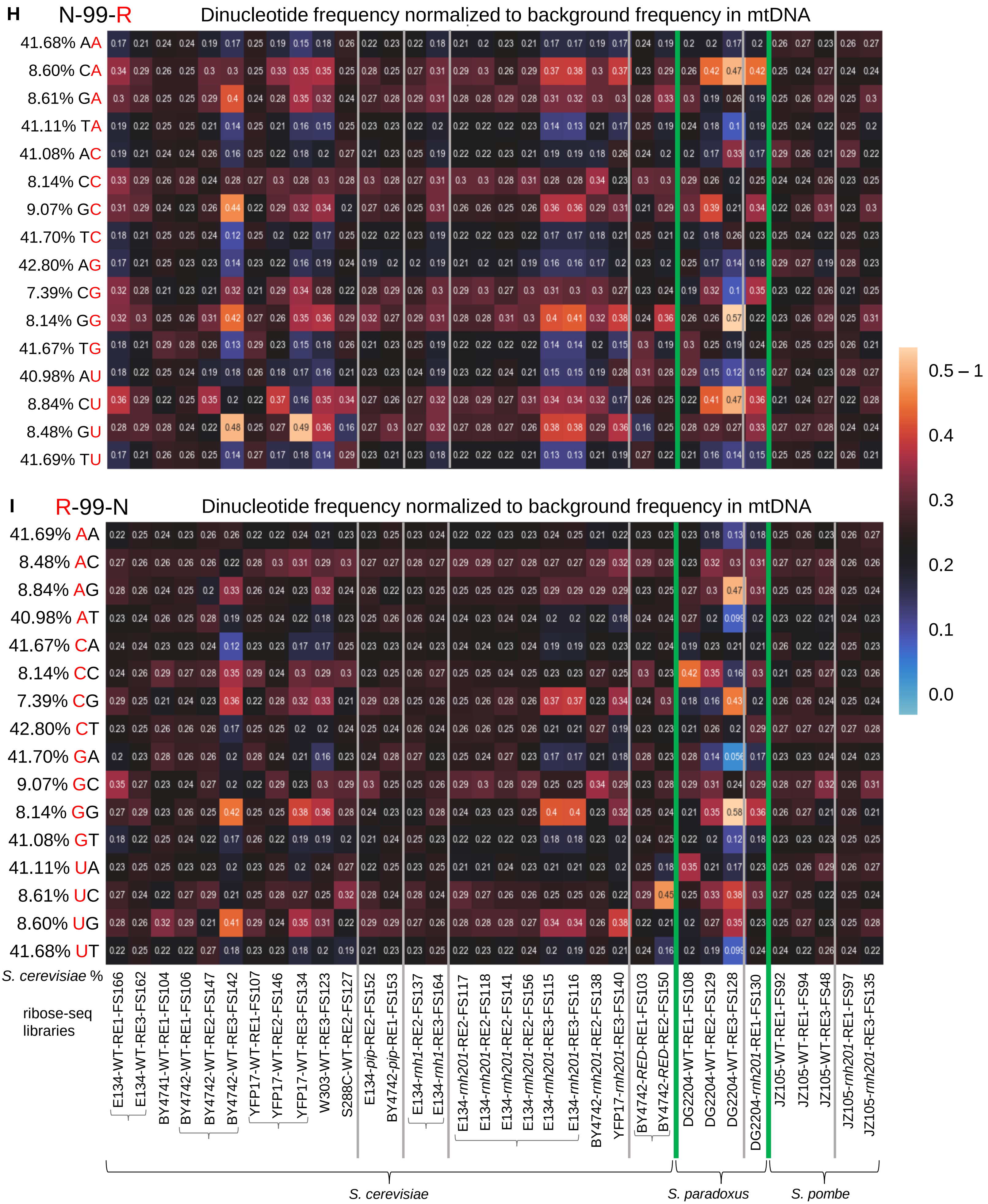
H
N-99-R
Dinucleotide frequency normalized to background frequency in mtDNA
41.68% AA
8.60% CA
8.61% GA
41.11% TA
41.08% AC
8.14% CC
9.07% GC
41.70% TC
42.80% AG
7.39% CG
8.14% GG
41.67% TG
40.98% AU
8.84% CU
8.48% GU
41.69% TU
I
R-99-N
Dinucleotide frequency normalized to background frequency in mtDNA
41.69% AA
8.48% AC
8.84% AG
40.98% AT
41.67% CA
8.14% CC
7.39% CG
42.80% CT
41.70% GA
9.07% GC
8.14% GG
41.08% GT
41.11% UA
8.61% UC
8.60% UG
41.68% UT
0.5 – 1
0.4
0.3
0.2
0.1
0.0
S. cerevisiae %
ribose-seq libraries
E134-WT-RE1-FS166
E134-WT-RE3-FS162
BY4741-WT-RE1-FS104
BY4742-WT-RE1-FS106
BY4742-WT-RE2-FS147
BY4742-WT-RE3-FS142
YFP17-WT-RE1-FS107
YFP17-WT-RE2-FS146
YFP17-WT-RE3-FS134
W303-WT-RE3-FS123
S288C-WT-RE2-FS127
E134-pip-RE2-FS152
BY4742-pip-RE1-FS153
E134-rnh1-RE2-FS137
E134-rnh1-RE3-FS164
E134-rnh201-RE2-FS117
E134-rnh201-RE2-FS118
E134-rnh201-RE2-FS141
E134-rnh201-RE2-FS156
E134-rnh201-RE3-FS115
E134-rnh201-RE3-FS116
BY4742-rnh201-RE2-FS138
YFP17-rnh201-RE3-FS140
BY4742-RED-RE1-FS103
BY4742-RED-RE2-FS150
DG2204-WT-RE1-FS108
DG2204-WT-RE2-FS129
DG2204-WT-RE3-FS128
DG2204-rnh201-RE1-FS130
JZ105-WT-RE1-FS92
JZ105-WT-RE1-FS94
JZ105-WT-RE3-FS48
JZ105-rnh201-RE1-FS97
JZ105-rnh201-RE3-FS135
S. cerevisiae
S. paradoxus
S. pombe

Supplementary Figure 3. dNMP positions having low impact on rNMP occurrence in yeast mtDNA. Heatmap analyses with normalized frequency of (**A**) RN, (**B**) N-R, (**C**) R-N, (**D**) N--R, (**E**) R--N, (**F**) N---R, (**G**) R---N, (**H**) N-99-R, and (**I**) R-99-N dinucleotides (rA, rC, rG and rU with the +1, -2, +2, -3, +3, -4, +4, -100 or +100 deoxyribonucleotide with base A, C, G or T) for all the mitochondrial ribose-seq libraries of this study. The formulas used to calculate these normalized frequencies are shown and explained in Methods. Each column of the heatmaps shows results of a specific ribose-seq library. Each library name is indicated underneath each column of the heatmaps with its corresponding strain name, genotype, and restriction-enzyme (RE) set used. The yeast species of the ribose-seq libraries are also indicated. *S. cerevisiae* libraries derived from the same strains are grouped together by curly brackets. Thick, vertical green lines separate data from the different yeast species. Vertical gray lines separate data obtained from different RNase H genotypes within each species. Each row shows results obtained for a dinucleotide RN (R in red), N-R, R-N, N--R, R--N, N---R, R---N, N-99-R, or R-99-N of fixed rNMP base A, C, G or U for each library. The actual % of dinucleotides of fixed base A, C, G or T for the indicated base combinations that are present in mtDNA of *S. cerevisiae* are shown to the left of the heatmaps and are also indicated in Supplementary Data 2A. The corresponding base % for mtDNA of *S. paradoxus* and *pombe* are indicated in Supplementary Data 2B and 2C, respectively. The observed % of dinucleotides with NMPs with base A, C, G or U were divided by the actual % of each dinucleotide with fixed base A, C, G or T in mtDNA of the corresponding species. The bar to the right shows how different frequency values are represented as different colors: black for 0.25; black to yellow for 0.25 to 0.5-1, and black to light blue for 0.25 to 0.

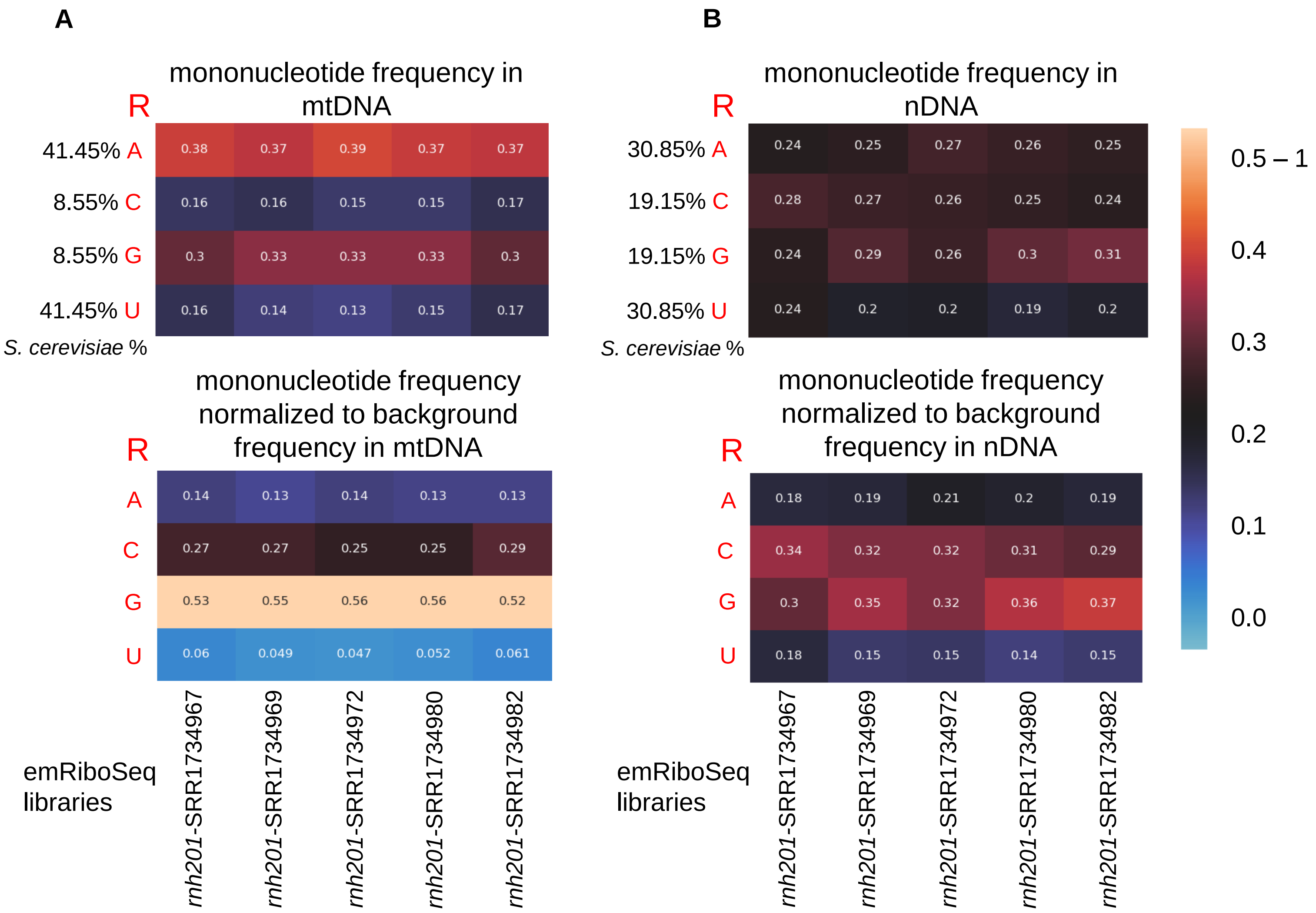
A
mononucleotide frequency in mtDNA
R
41.45% A
8.55% C
8.55% G
41.45% U
S. cerevisiae %
0.38 0.37 0.39 0.37 0.37
0.16 0.16 0.15 0.15 0.17
0.3 0.33 0.33 0.33 0.3
0.16 0.14 0.13 0.15 0.17
mononucleotide frequency normalized to background frequency in mtDNA
R
A
C
G
U
0.14 0.13 0.14 0.13 0.13
0.27 0.27 0.25 0.25 0.29
0.53 0.55 0.56 0.56 0.52
0.06 0.049 0.047 0.052 0.061
emRiboSeq libraries
rnh201-SRR1734967
rnh201-SRR1734969
rnh201-SRR1734972
rnh201-SRR1734980
rnh201-SRR1734982
B
mononucleotide frequency in nDNA
R
30.85% A
19.15% C
19.15% G
30.85% U
S. cerevisiae %
0.24 0.25 0.27 0.26 0.25
0.28 0.27 0.26 0.25 0.24
0.24 0.29 0.26 0.3 0.31
0.24 0.2 0.2 0.19 0.2
mononucleotide frequency normalized to background frequency in nDNA
R
A
C
G
U
0.18 0.19 0.21 0.2 0.19
0.34 0.32 0.32 0.31 0.29
0.3 0.35 0.32 0.36 0.37
0.18 0.15 0.15 0.14 0.15
emRiboSeq libraries
rnh201-SRR1734967
rnh201-SRR1734969
rnh201-SRR1734972
rnh201-SRR1734980
rnh201-SRR1734982
0.5 – 1
0.4
0.3
0.2
0.1
0.0

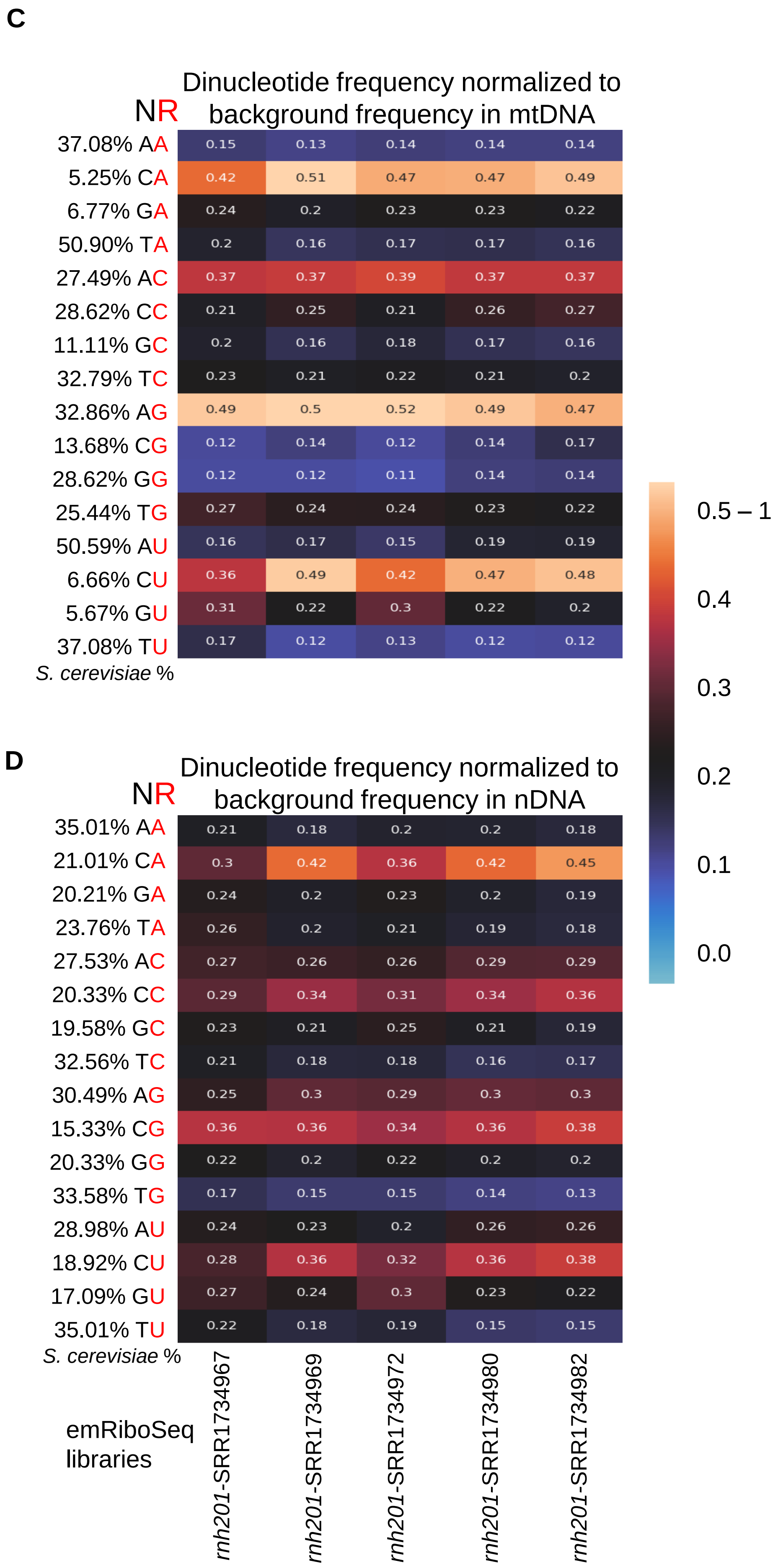
C
Dinucleotide frequency normalized to background frequency in mtDNA
NR
37.08% AA 0.15 0.13 0.14 0.14 0.14
5.25% CA 0.42 0.51 0.47 0.47 0.49
6.77% GA 0.24 0.2 0.23 0.23 0.22
50.90% TA 0.2 0.16 0.17 0.17 0.16
27.49% AC 0.37 0.37 0.39 0.37 0.37
28.62% CC 0.21 0.25 0.21 0.26 0.27
11.11% GC 0.2 0.16 0.18 0.17 0.16
32.79% TC 0.23 0.21 0.22 0.21 0.2
32.86% AG 0.49 0.5 0.52 0.49 0.47
13.68% CG 0.12 0.14 0.12 0.14 0.17
28.62% GG 0.12 0.12 0.11 0.14 0.14
25.44% TG 0.27 0.24 0.24 0.23 0.22
50.59% AU 0.16 0.17 0.15 0.19 0.19
6.66% CU 0.36 0.49 0.42 0.47 0.48
5.67% GU 0.31 0.22 0.3 0.22 0.2
37.08% TU 0.17 0.12 0.13 0.12 0.12
S. cerevisiae %
0.5 – 1
0.4
0.3
0.2
0.1
0.0
D
Dinucleotide frequency normalized to background frequency in nDNA
NR
35.01% AA 0.21 0.18 0.2 0.2 0.18
21.01% CA 0.3 0.42 0.36 0.42 0.45
20.21% GA 0.24 0.2 0.23 0.2 0.19
23.76% TA 0.26 0.2 0.21 0.19 0.18
27.53% AC 0.27 0.26 0.26 0.29 0.29
20.33% CC 0.29 0.34 0.31 0.34 0.36
19.58% GC 0.23 0.21 0.25 0.21 0.19
32.56% TC 0.21 0.18 0.18 0.16 0.17
30.49% AG 0.25 0.3 0.29 0.3 0.3
15.33% CG 0.36 0.36 0.34 0.36 0.38
20.33% GG 0.22 0.2 0.22 0.2 0.2
33.58% TG 0.17 0.15 0.15 0.14 0.13
28.98% AU 0.24 0.23 0.2 0.26 0.26
18.92% CU 0.28 0.36 0.32 0.36 0.38
17.09% GU 0.27 0.24 0.3 0.23 0.22
35.01% TU 0.22 0.18 0.19 0.15 0.15
S. cerevisiae %
emRiboSeq libraries
rnh201-SRR1734967
rnh201-SRR1734969
rnh201-SRR1734972
rnh201-SRR1734980
rnh201-SRR1734982

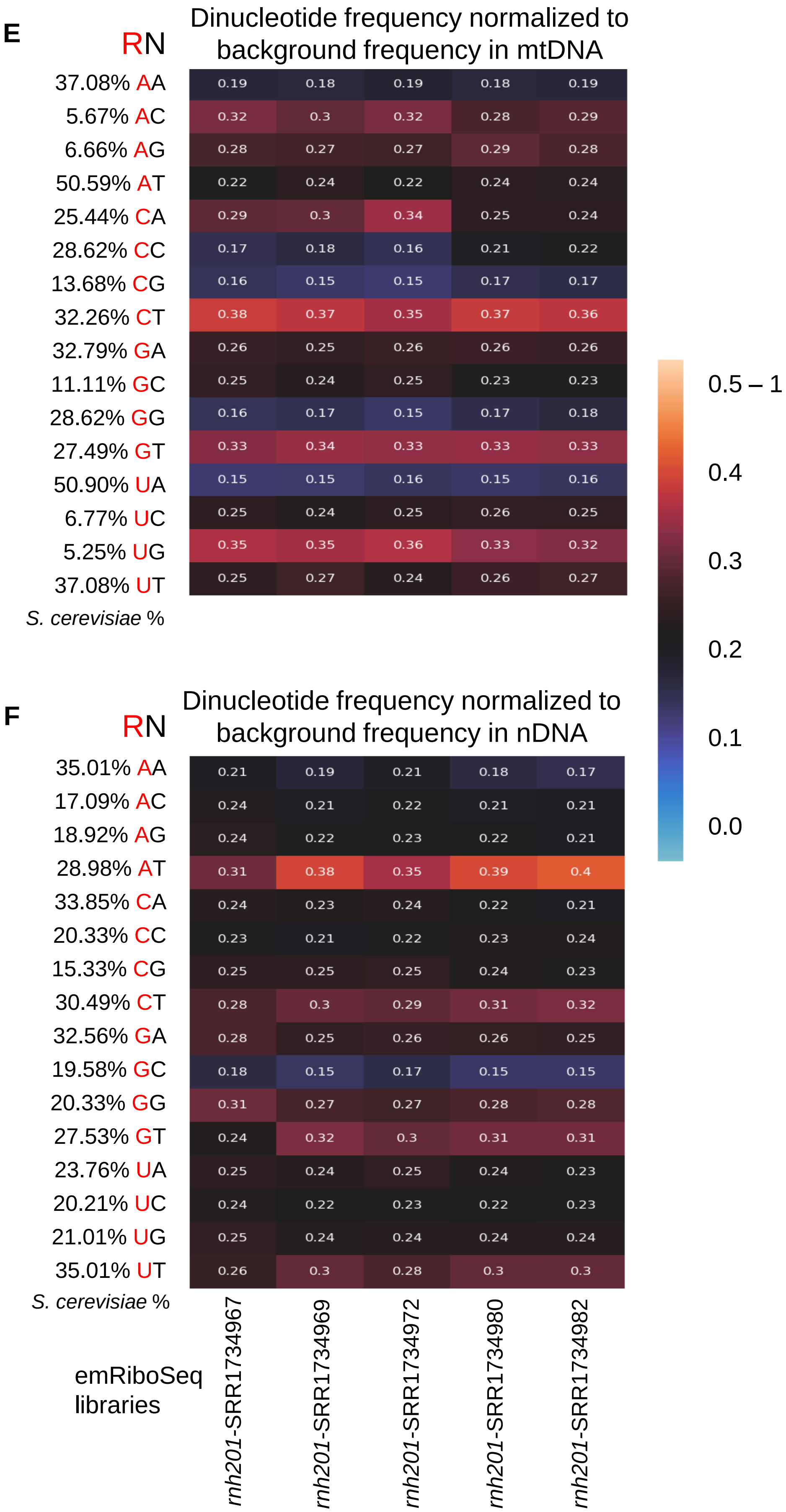

E
RN
Dinucleotide frequency normalized to background frequency in mtDNA
37.08% AA 0.19 0.18 0.19 0.18 0.19
5.67% AC 0.32 0.3 0.32 0.28 0.29
6.66% AG 0.28 0.27 0.27 0.29 0.28
50.59% AT 0.22 0.24 0.22 0.24 0.24
25.44% CA 0.29 0.3 0.34 0.25 0.24
28.62% CC 0.17 0.18 0.16 0.21 0.22
13.68% CG 0.16 0.15 0.15 0.17 0.17
32.26% CT 0.38 0.37 0.35 0.37 0.36
32.79% GA 0.26 0.25 0.26 0.26 0.26
11.11% GC 0.25 0.24 0.25 0.23 0.23
28.62% GG 0.16 0.17 0.15 0.17 0.18
27.49% GT 0.33 0.34 0.33 0.33 0.33
50.90% UA 0.15 0.15 0.16 0.15 0.16
6.77% UC 0.25 0.24 0.25 0.26 0.25
5.25% UG 0.35 0.35 0.36 0.33 0.32
37.08% UT 0.25 0.27 0.24 0.26 0.27
S. cerevisiae %
F
RN
Dinucleotide frequency normalized to background frequency in nDNA
35.01% AA 0.21 0.19 0.21 0.18 0.17
17.09% AC 0.24 0.21 0.22 0.21 0.21
18.92% AG 0.24 0.22 0.23 0.22 0.21
28.98% AT 0.31 0.38 0.35 0.39 0.4
33.85% CA 0.24 0.23 0.24 0.22 0.21
20.33% CC 0.23 0.21 0.22 0.23 0.24
15.33% CG 0.25 0.25 0.25 0.24 0.23
30.49% CT 0.28 0.3 0.29 0.31 0.32
32.56% GA 0.28 0.25 0.26 0.26 0.25
19.58% GC 0.18 0.15 0.17 0.15 0.15
20.33% GG 0.31 0.27 0.27 0.28 0.28
27.53% GT 0.24 0.32 0.3 0.31 0.31
23.76% UA 0.25 0.24 0.25 0.24 0.23
20.21% UC 0.24 0.22 0.23 0.22 0.23
21.01% UG 0.25 0.24 0.24 0.24 0.24
35.01% UT 0.26 0.3 0.28 0.3 0.3
S. cerevisiae %
emRiboSeq libraries
rnh201-SRR1734967
rnh201-SRR1734969
rnh201-SRR1734972
rnh201-SRR1734980
rnh201-SRR1734982
0.5 – 1
0.4
0.3
0.2
0.1
0.0

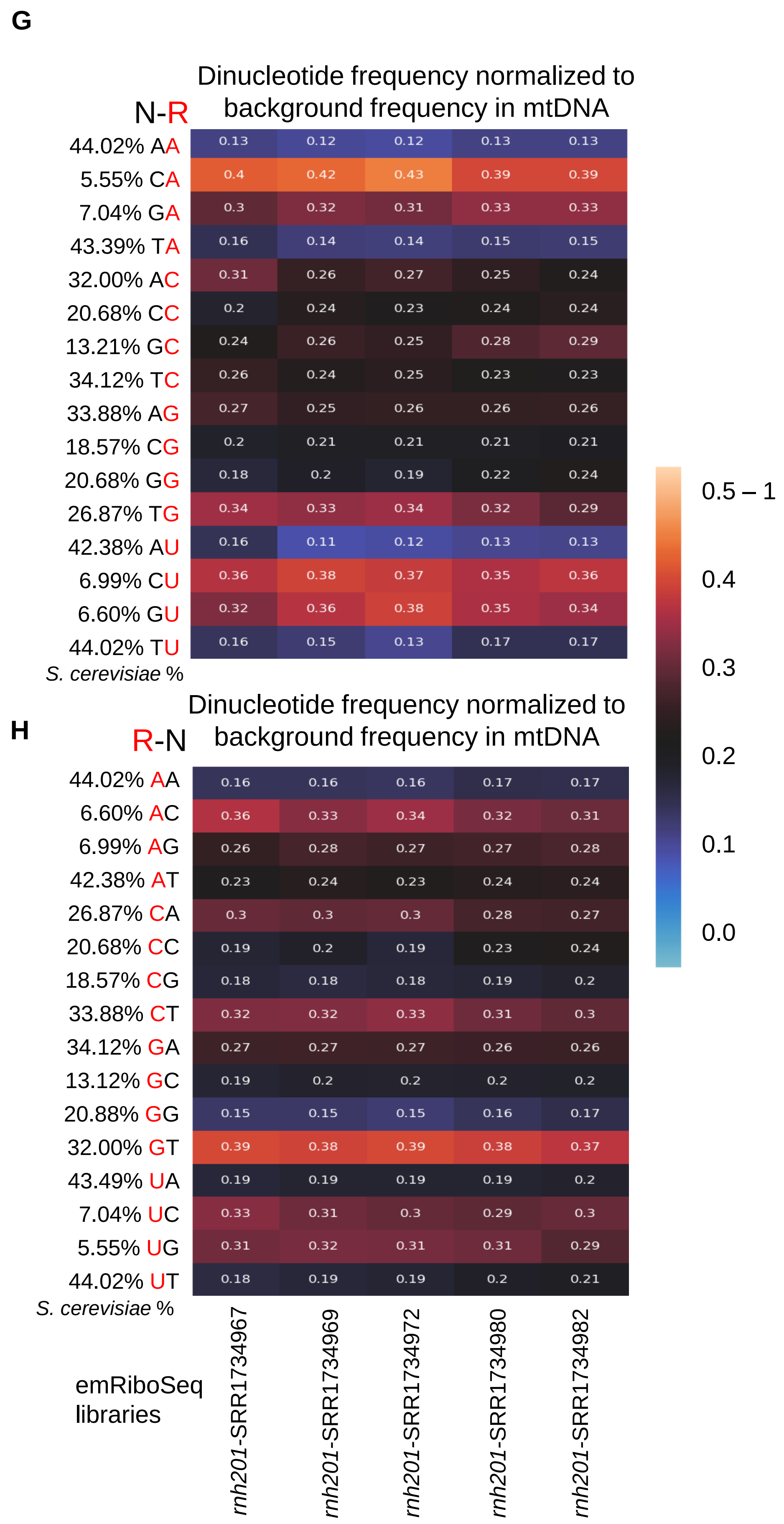
G
Dinucleotide frequency normalized to background frequency in mtDNA
N-R
44.02% AA 0.13 0.12 0.12 0.13 0.13
5.55% CA 0.4 0.42 0.43 0.39 0.39
7.04% GA 0.3 0.32 0.31 0.33 0.33
43.39% TA 0.16 0.14 0.14 0.15 0.15
32.00% AC 0.31 0.26 0.27 0.25 0.24
20.68% CC 0.2 0.24 0.23 0.24 0.24
13.21% GC 0.24 0.26 0.25 0.28 0.29
34.12% TC 0.26 0.24 0.25 0.23 0.23
33.88% AG 0.27 0.25 0.26 0.26 0.26
18.57% CG 0.2 0.21 0.21 0.21 0.21
20.68% GG 0.18 0.2 0.19 0.22 0.24
26.87% TG 0.34 0.33 0.34 0.32 0.29
42.38% AU 0.16 0.11 0.12 0.13 0.13
6.99% CU 0.36 0.38 0.37 0.35 0.36
6.60% GU 0.32 0.36 0.38 0.35 0.34
44.02% TU 0.16 0.15 0.13 0.17 0.17
S. cerevisiae %
H
Dinucleotide frequency normalized to background frequency in mtDNA
R-N
44.02% AA 0.16 0.16 0.16 0.17 0.17
6.60% AC 0.36 0.33 0.34 0.32 0.31
6.99% AG 0.26 0.28 0.27 0.27 0.28
42.38% AT 0.23 0.24 0.23 0.24 0.24
26.87% CA 0.3 0.3 0.3 0.28 0.27
20.68% CC 0.19 0.2 0.19 0.23 0.24
18.57% CG 0.18 0.18 0.18 0.19 0.2
33.88% CT 0.32 0.32 0.33 0.31 0.3
34.12% GA 0.27 0.27 0.27 0.26 0.26
13.12% GC 0.19 0.2 0.2 0.2 0.2
20.88% GG 0.15 0.15 0.15 0.16 0.17
32.00% GT 0.39 0.38 0.39 0.38 0.37
43.49% UA 0.19 0.19 0.19 0.19 0.2
7.04% UC 0.33 0.31 0.3 0.29 0.3
5.55% UG 0.31 0.32 0.31 0.31 0.29
44.02% UT 0.18 0.19 0.19 0.2 0.21
S. cerevisiae %
emRiboSeq libraries
rnh201-SRR1734967
rnh201-SRR1734969
rnh201-SRR1734972
rnh201-SRR1734980
rnh201-SRR1734982
0.5 – 1
0.4
0.3
0.2
0.1
0.0

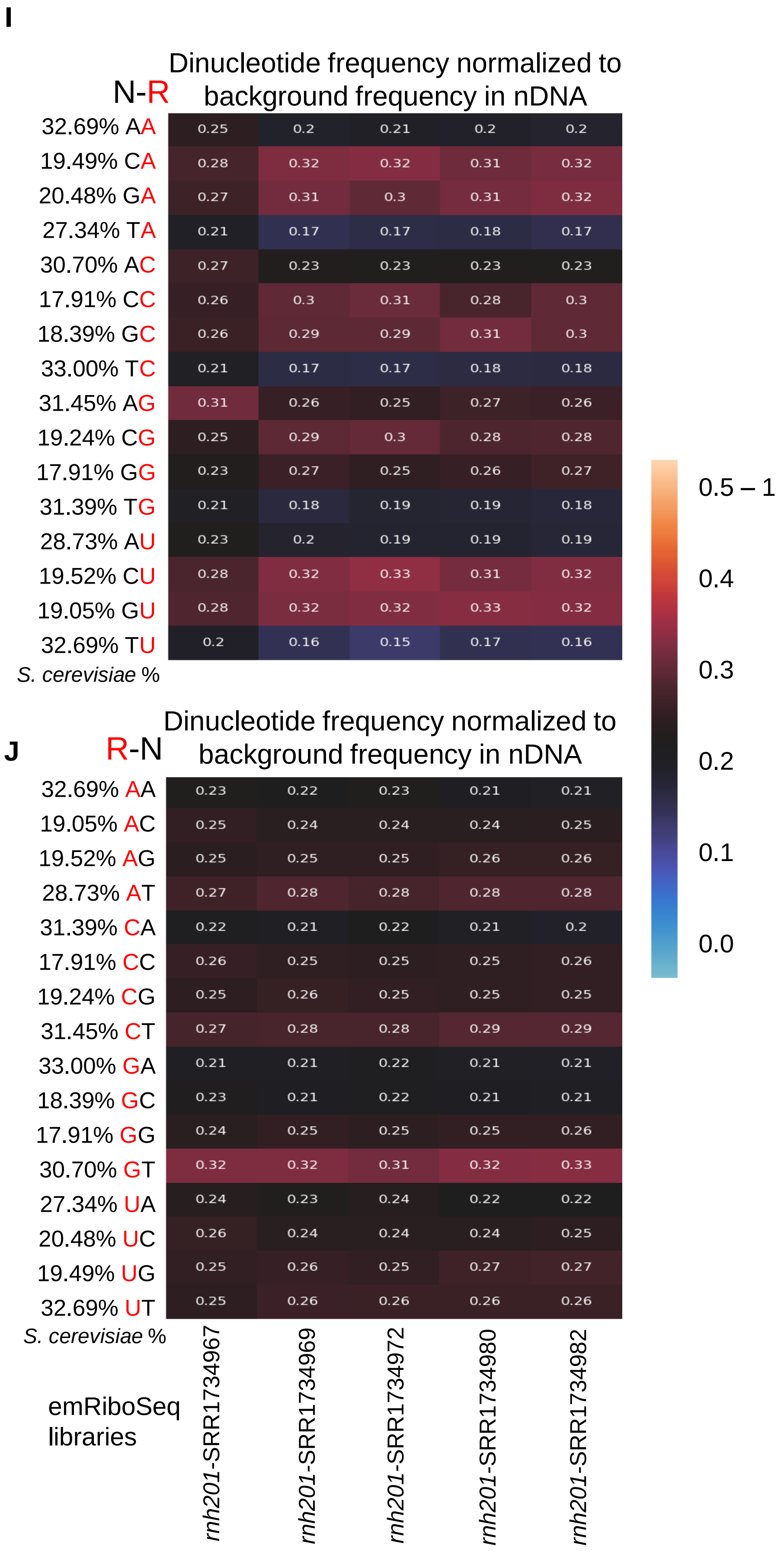
I
N-R
Dinucleotide frequency normalized to background frequency in nDNA
32.69% AA 0.25 0.2 0.21 0.2 0.2
19.49% CA 0.28 0.32 0.32 0.31 0.32
20.48% GA 0.27 0.31 0.3 0.31 0.32
27.34% TA 0.21 0.17 0.17 0.18 0.17
30.70% AC 0.27 0.23 0.23 0.23 0.23
17.91% CC 0.26 0.3 0.31 0.28 0.3
18.39% GC 0.26 0.29 0.29 0.31 0.3
33.00% TC 0.21 0.17 0.17 0.18 0.18
31.45% AG 0.31 0.26 0.25 0.27 0.26
19.24% CG 0.25 0.29 0.3 0.28 0.28
17.91% GG 0.23 0.27 0.25 0.26 0.27
31.39% TG 0.21 0.18 0.19 0.19 0.18
28.73% AU 0.23 0.2 0.19 0.19 0.19
19.52% CU 0.28 0.32 0.33 0.31 0.32
19.05% GU 0.28 0.32 0.32 0.33 0.32
32.69% TU 0.2 0.16 0.15 0.17 0.16
S. cerevisiae %
J
R-N
Dinucleotide frequency normalized to background frequency in nDNA
32.69% AA 0.23 0.22 0.23 0.21 0.21
19.05% AC 0.25 0.24 0.24 0.24 0.25
19.52% AG 0.25 0.25 0.25 0.26 0.26
28.73% AT 0.27 0.28 0.28 0.28 0.28
31.39% CA 0.22 0.21 0.22 0.21 0.2
17.91% CC 0.26 0.25 0.25 0.25 0.26
19.24% CG 0.25 0.26 0.25 0.25 0.25
31.45% CT 0.27 0.28 0.28 0.29 0.29
33.00% GA 0.21 0.21 0.22 0.21 0.21
18.39% GC 0.23 0.21 0.22 0.21 0.21
17.91% GG 0.24 0.25 0.25 0.25 0.26
30.70% GT 0.32 0.32 0.31 0.32 0.33
27.34% UA 0.24 0.23 0.24 0.22 0.22
20.48% UC 0.26 0.24 0.24 0.24 0.25
19.49% UG 0.25 0.26 0.25 0.27 0.27
32.69% UT 0.25 0.26 0.26 0.26 0.26
S. cerevisiae %
emRiboSeq libraries
rnh201-SRR1734967
rnh201-SRR1734969
rnh201-SRR1734972
rnh201-SRR1734980
rnh201-SRR1734982
0.5 – 1
0.4
0.3
0.2
0.1
0.0

Supplementary Figure 4. Impact of dNMP positions on rNMP occurrence in mtDNA and nDNA of emRiboSeq libraries. Heatmap analyses with (top) frequency of each type of rNMP (rA, rC, rG and rU), and (bottom) frequency of each type of rNMP normalized to the nucleotide frequencies of the corresponding reference genome for all the (**A**) mitochondrial and (**B**) nuclear emRiboSeq libraries used in the Reijns *et al.,* 2015 study. Heatmap analyses with normalized frequency of (**C**) NR, (**E**) RN, (**G**) N-R and (**H**) R-N for mitochondrial emRiboSeq libraries, and (**D**) NR, (**F**) RN, (**I**) N-R and (**J**) R-N for nuclear emRiboSeq libraries. The observed % of dinucleotides with NMPs with base A, C, G or U were divided by the actual % of each dinucleotide with fixed base A, C, G or T in mtDNA of the *S. cerevisiae*. The formulas used to calculate these normalized frequencies are shown and explained in Methods. Each column of the heatmaps shows results of a specific emRiboSeq library. The library name is indicated underneath each column of the heatmaps with its corresponding strain name that was used. The observed % of dinucleotides with NMPs with base A, C, G or U were divided by the actual % of each dinucleotide with fixed base A, C, G or T in mtDNA of the *S. cerevisiae*. The bar to the right shows how different frequency values are represented as different colors: black for 0.25; black to yellow for 0.25 to 0.5-1, and black to light blue for 0.25 to 0.

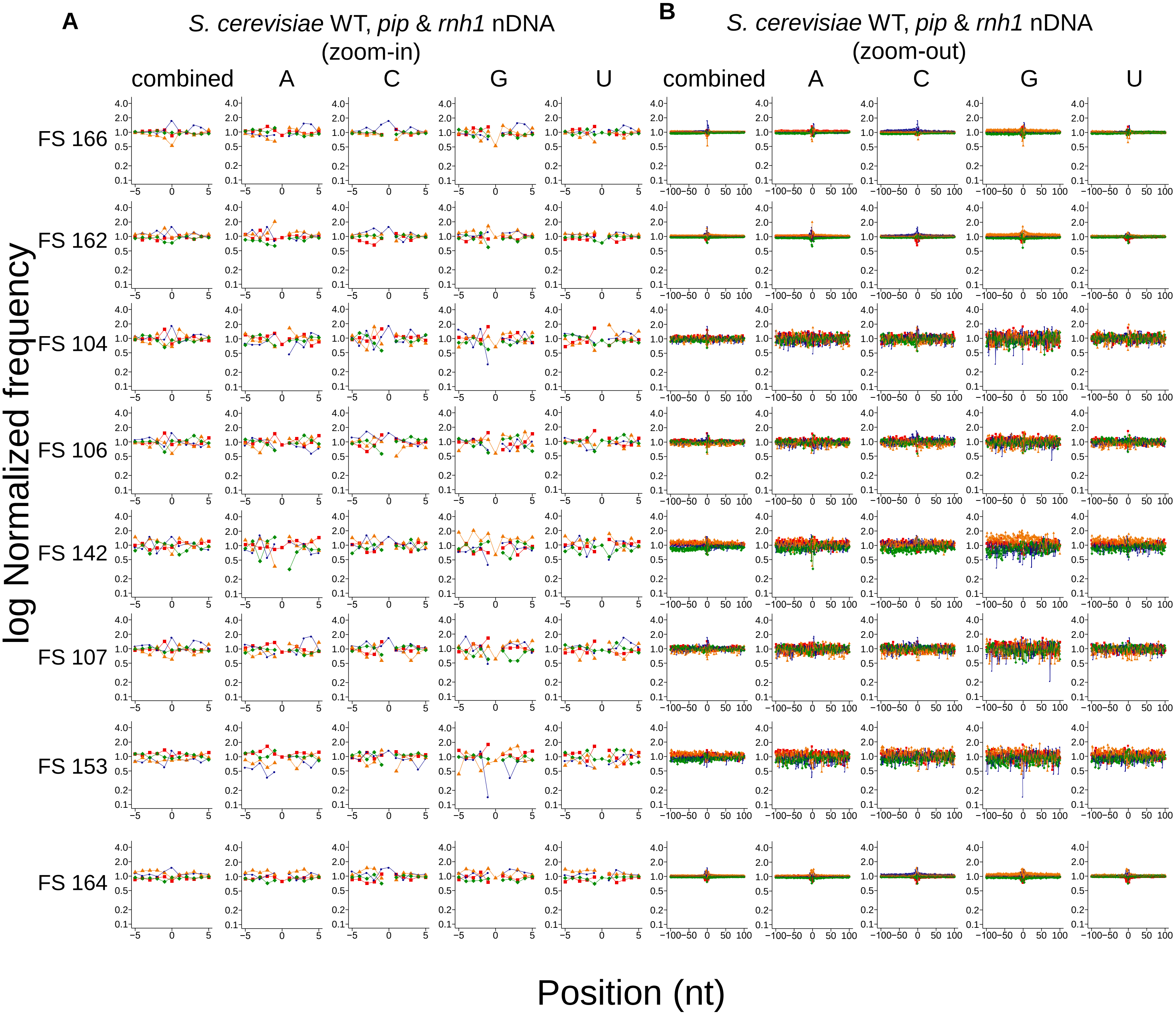

A
S. cerevisiae WT, pip & rnh1 nDNA (zoom-in)
B
S. cerevisiae WT, pip & rnh1 nDNA (zoom-out)
combined
A
C
G
U
FS 166
FS 162
FS 104
FS 106
FS 142
FS 107
FS 153
FS 164
log Normalized frequency
Position (nt)

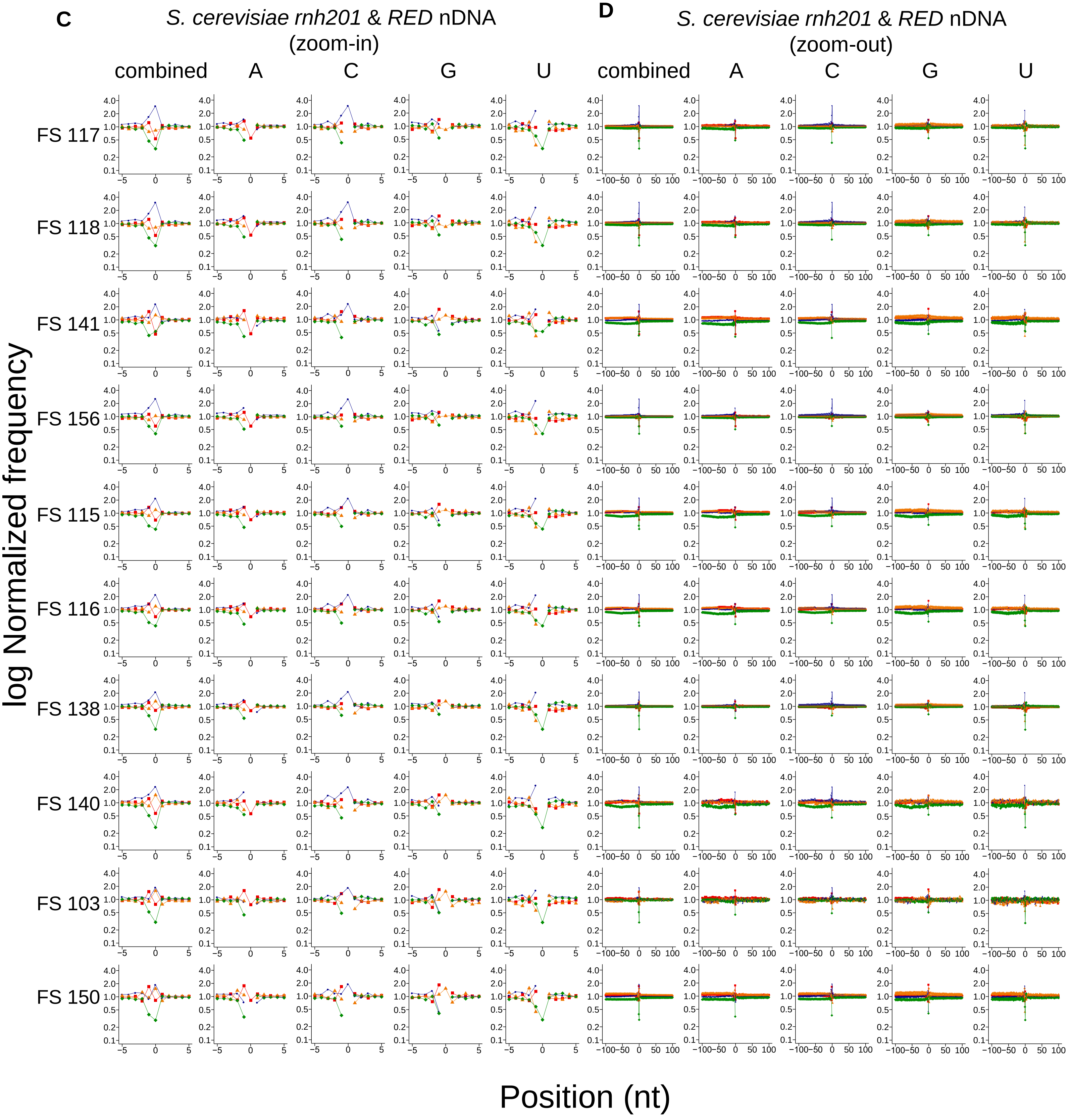
C
S. cerevisiae rnh201 & RED nDNA
(zoom-in)
D
S. cerevisiae rnh201 & RED nDNA
(zoom-out)
combined
A
C
G
U
FS 117
FS 118
FS 141
FS 156
FS 115
FS 116
FS 138
FS 140
FS 103
FS 150
log Normalized frequency
Position (nt)

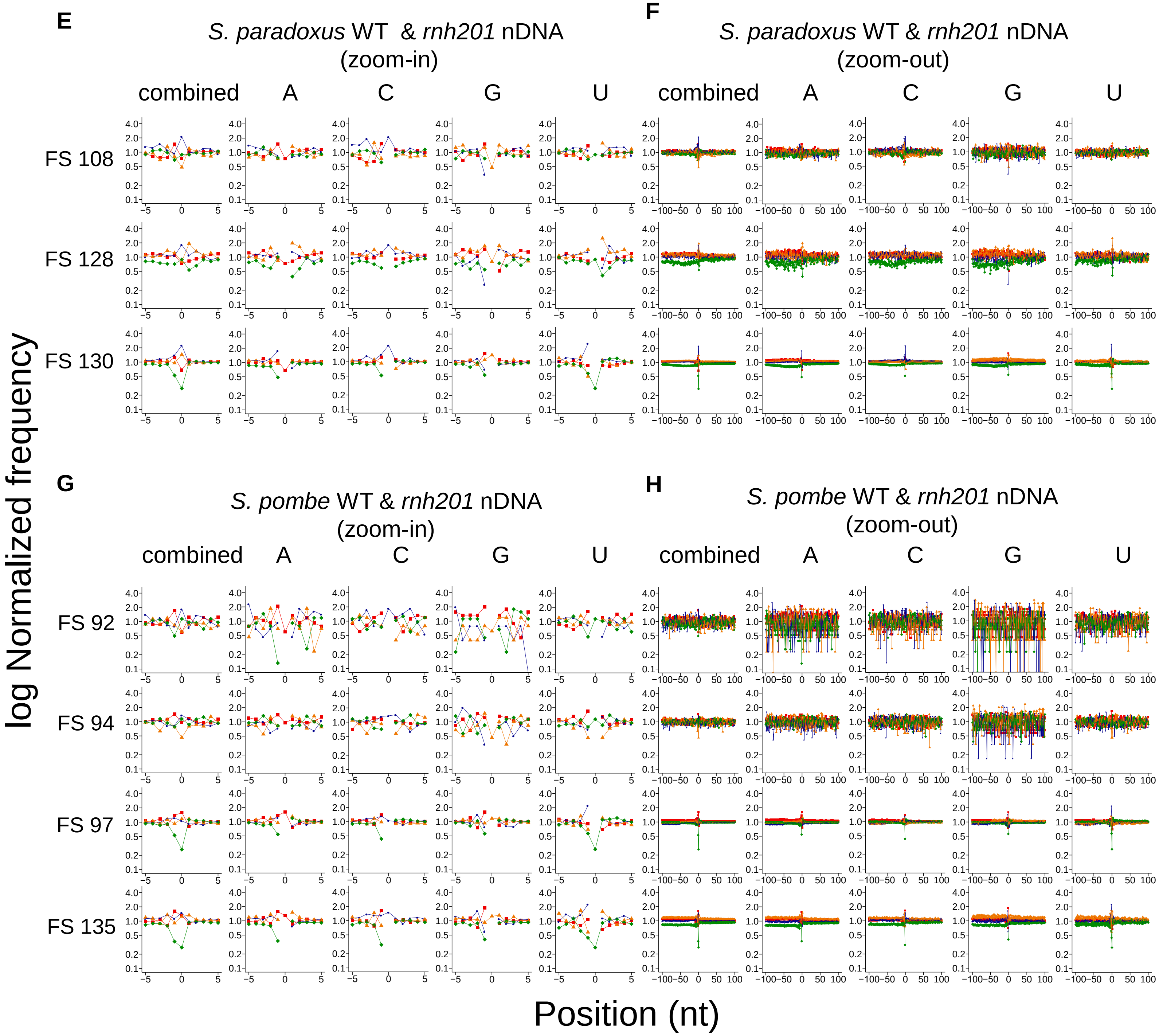

Supplementary Figure 5. Nucleotide plots from all nuclear libraries. (**A-H**) Plots of normalized nucleotide frequencies relative to mapped positions of sequences from the (**A,B**) 6 wild-type, 1 *pip* and 1 *rnh1 S. cerevisiae* nuclear libraries generated in this study, combined and single, (**A**) zoom-in, and (**B**) zoomed-out plots; (**C,D**) 8 *rnh201* and 2 *RED S. cerevisiae* nuclear libraries combined and single, (**C**) zoom-in, and (**D**) zoomed-out plots; (**E,F**) 2 *S. paradoxus* wild-type and 1 *rnh201* nuclear libraries combined and single, (**E**) zoom-in, and (**F**) zoomed-out plots; (**G,H**) 2 *S. pombe* wild-type and 2 *rnh201* nuclear libraries combined and single, (**G**) zoom-in, and (**H**) zoomed-out plots. Position 0 on the x-axis represents the site of rNMP incorporation, - and + positions represent upstream and downstream dNMPs, respectively. The y-axis shows the frequency of each type of nucleotide present in the ribose-seq data normalized to the frequency of the corresponding nucleotide present in the reference genome of the indicted yeast species. Red square, A; blue circle, C; orange triangle, G; and green rhombus, U.

A

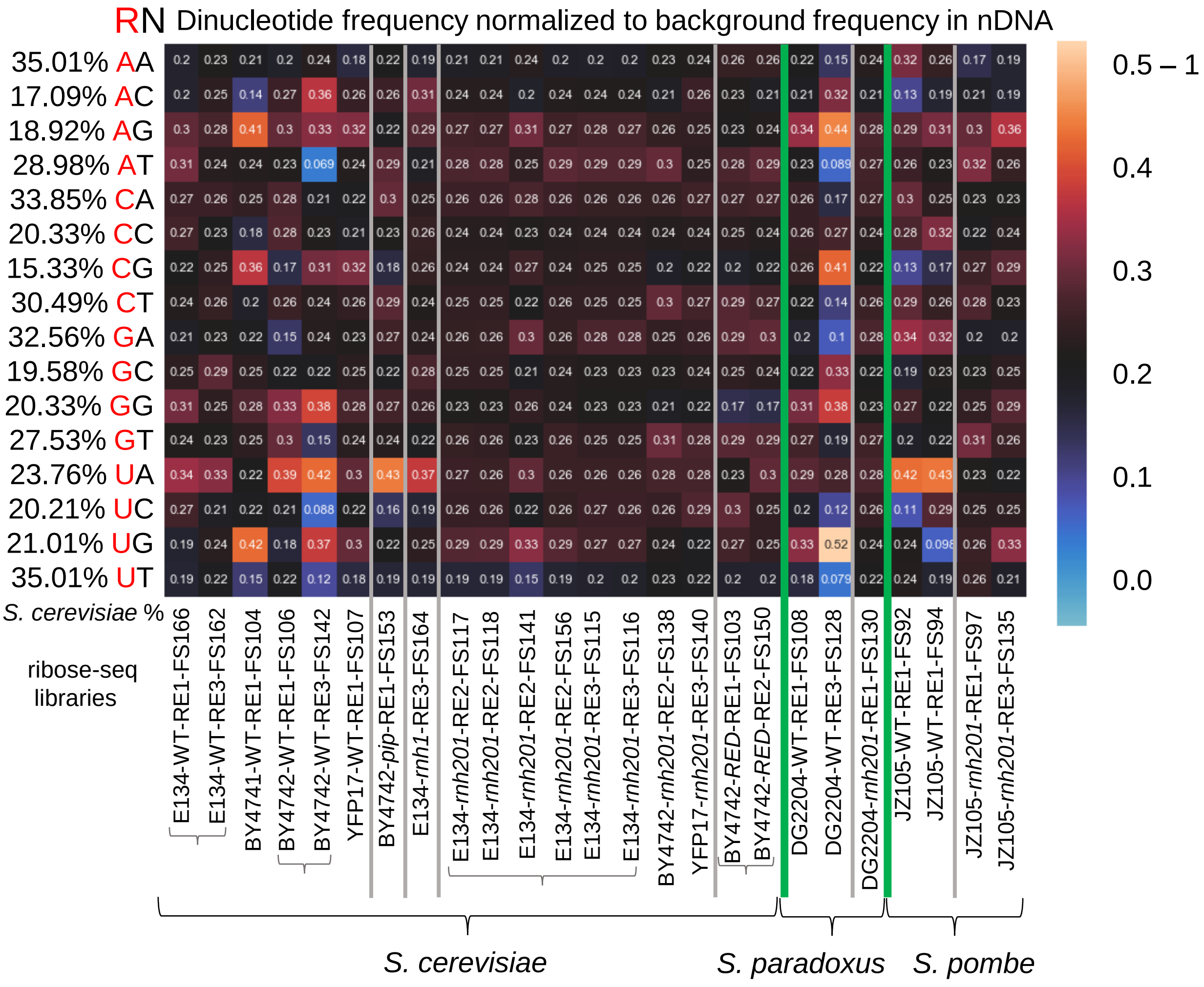
RN Dinucleotide frequency normalized to background frequency in nDNA
35.01% AA
17.09% AC
18.92% AG
28.98% AT
33.85% CA
20.33% CC
15.33% CG
30.49% CT
32.56% GA
19.58% GC
20.33% GG
27.53% GT
23.76% UA
20.21% UC
21.01% UG
35.01% UT
S. cerevisiae %
ribose-seq libraries
E134-WT-RE1-FS166
E134-WT-RE3-FS162
BY4741-WT-RE1-FS104
BY4742-WT-RE1-FS106
BY4742-WT-RE3-FS142
YFP17-WT-RE1-FS107
BY4742-pip-RE1-FS153
E134-rnh1-RE3-FS164
E134-rnh201-RE2-FS117
E134-rnh201-RE2-FS118
E134-rnh201-RE2-FS141
E134-rnh201-RE2-FS156
E134-rnh201-RE3-FS115
E134-rnh201-RE3-FS116
BY4742-rnh201-RE2-FS138
YFP17-rnh201-RE3-FS140
BY4742-RED-RE1-FS103
BY4742-RED-RE2-FS150
DG2204-WT-RE1-FS108
DG2204-WT-RE3-FS128
DG2204-rnh201-RE1-FS130
JZ105-WT-RE1-FS92
JZ105-WT-RE1-FS94
JZ105-rnh201-RE1-FS97
JZ105-rnh201-RE3-FS135
S. cerevisiae
S. paradoxus
S. pombe
0.5 – 1
0.4
0.3
0.2
0.1
0.0

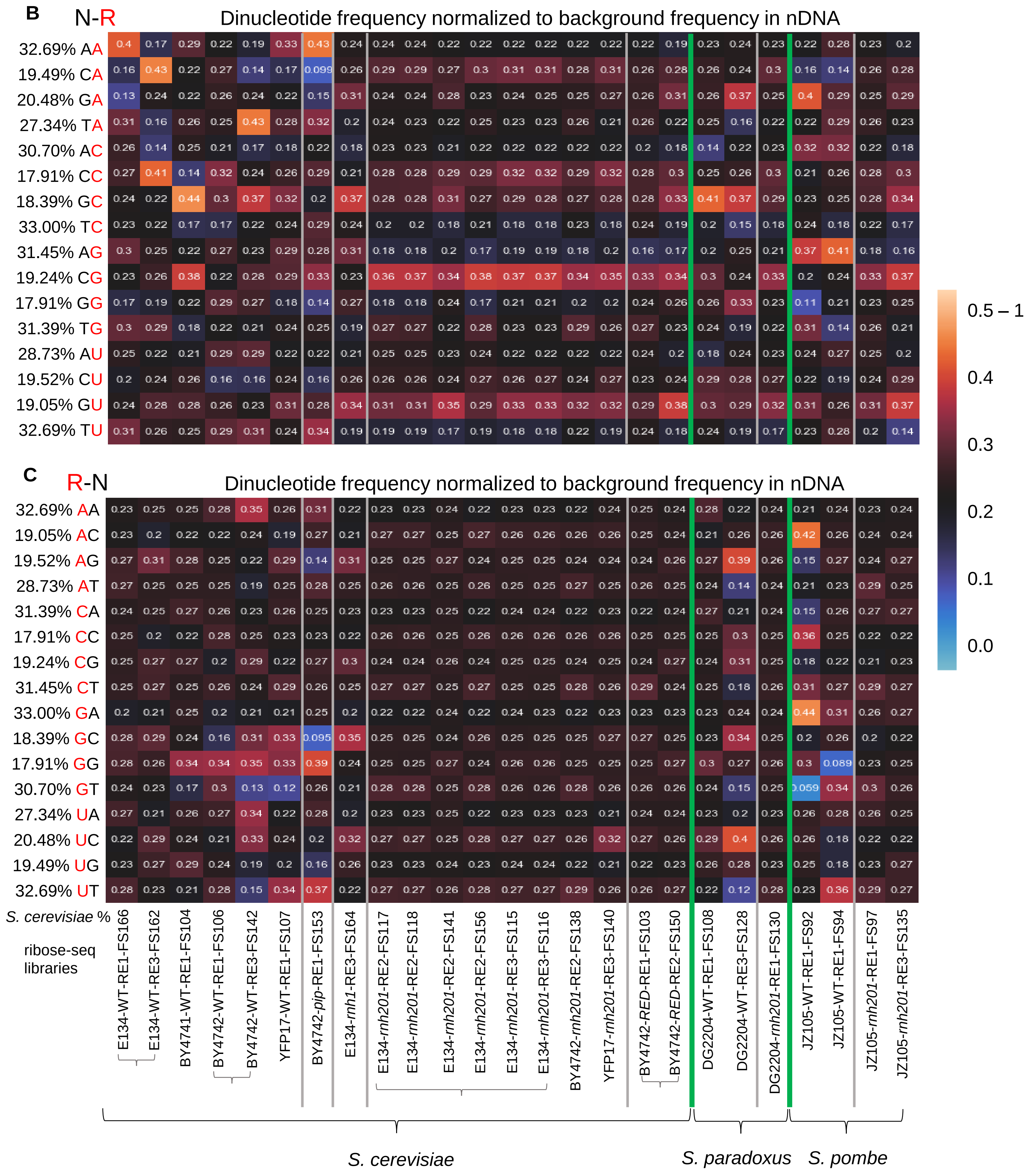

B
N-R
Dinucleotide frequency normalized to background frequency in nDNA
32.69% AA
19.49% CA
20.48% GA
27.34% TA
30.70% AC
17.91% CC
18.39% GC
33.00% TC
31.45% AG
19.24% CG
17.91% GG
31.39% TG
28.73% AU
19.52% CU
19.05% GU
32.69% TU
C
R-N
Dinucleotide frequency normalized to background frequency in nDNA
32.69% AA
19.05% AC
19.52% AG
28.73% AT
31.39% CA
17.91% CC
19.24% CG
31.45% CT
33.00% GA
18.39% GC
17.91% GG
30.70% GT
27.34% UA
20.48% UC
19.49% UG
32.69% UT
0.5 – 1
0.4
0.3
0.2
0.1
0.0
S. cerevisiae %
ribose-seq libraries
E134-WT-RE1-FS166
E134-WT-RE3-FS162
BY4741-WT-RE1-FS104
BY4742-WT-RE1-FS106
BY4742-WT-RE3-FS142
YFP17-WT-RE1-FS107
BY4742-pip-RE1-FS153
E134-rnh1-RE3-FS164
E134-rnh201-RE2-FS117
E134-rnh201-RE2-FS118
E134-rnh201-RE2-FS141
E134-rnh201-RE2-FS156
E134-rnh201-RE3-FS115
E134-rnh201-RE3-FS116
BY4742-rnh201-RE2-FS138
YFP17-rnh201-RE3-FS140
BY4742-RED-RE1-FS103
BY4742-RED-RE2-FS150
DG2204-WT-RE1-FS108
DG2204-WT-RE3-FS128
DG2204-rnh201-RE1-FS130
JZ105-WT-RE1-FS92
JZ105-WT-RE1-FS94
JZ105-rnh201-RE1-FS97
JZ105-rnh201-RE3-FS135
S. cerevisiae
S. paradoxus
S. pombe

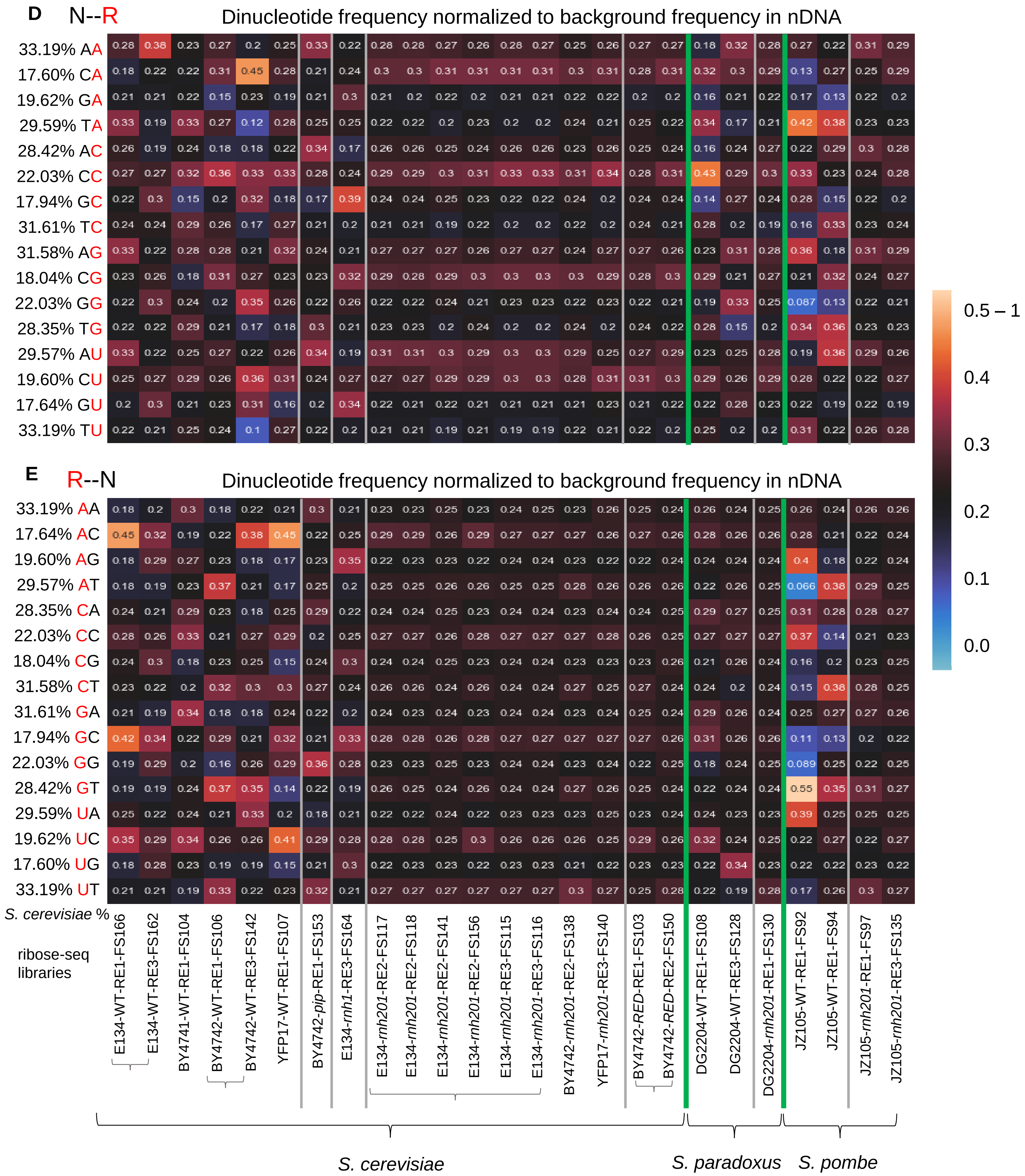
D
N--R
Dinucleotide frequency normalized to background frequency in nDNA
33.19% AA
17.60% CA
19.62% GA
29.59% TA
28.42% AC
22.03% CC
17.94% GC
31.61% TC
31.58% AG
18.04% CG
22.03% GG
28.35% TG
29.57% AU
19.60% CU
17.64% GU
33.19% TU
E
R--N
Dinucleotide frequency normalized to background frequency in nDNA
33.19% AA
17.64% AC
19.60% AG
29.57% AT
28.35% CA
22.03% CC
18.04% CG
31.58% CT
31.61% GA
17.94% GC
22.03% GG
28.42% GT
29.59% UA
19.62% UC
17.60% UG
33.19% UT
S. cerevisiae %
ribose-seq libraries
E134-WT-RE1-FS166
E134-WT-RE3-FS162
BY4741-WT-RE1-FS104
BY4742-WT-RE1-FS106
BY4742-WT-RE3-FS142
YFP17-WT-RE1-FS107
BY4742-pip-RE1-FS153
E134-rnh1-RE3-FS164
E134-rnh201-RE2-FS117
E134-rnh201-RE2-FS118
E134-rnh201-RE2-FS141
E134-rnh201-RE2-FS156
E134-rnh201-RE3-FS115
E134-rnh201-RE3-FS116
BY4742-rnh201-RE2-FS138
YFP17-rnh201-RE3-FS140
BY4742-RED-RE1-FS103
BY4742-RED-RE2-FS150
DG2204-WT-RE1-FS108
DG2204-WT-RE3-FS128
DG2204-rnh201-RE1-FS130
JZ105-WT-RE1-FS92
JZ105-WT-RE1-FS94
JZ105-rnh201-RE1-FS97
JZ105-rnh201-RE3-FS135
S. cerevisiae
S. paradoxus
S. pombe
0.5 – 1
0.4
0.3
0.2
0.1
0.0

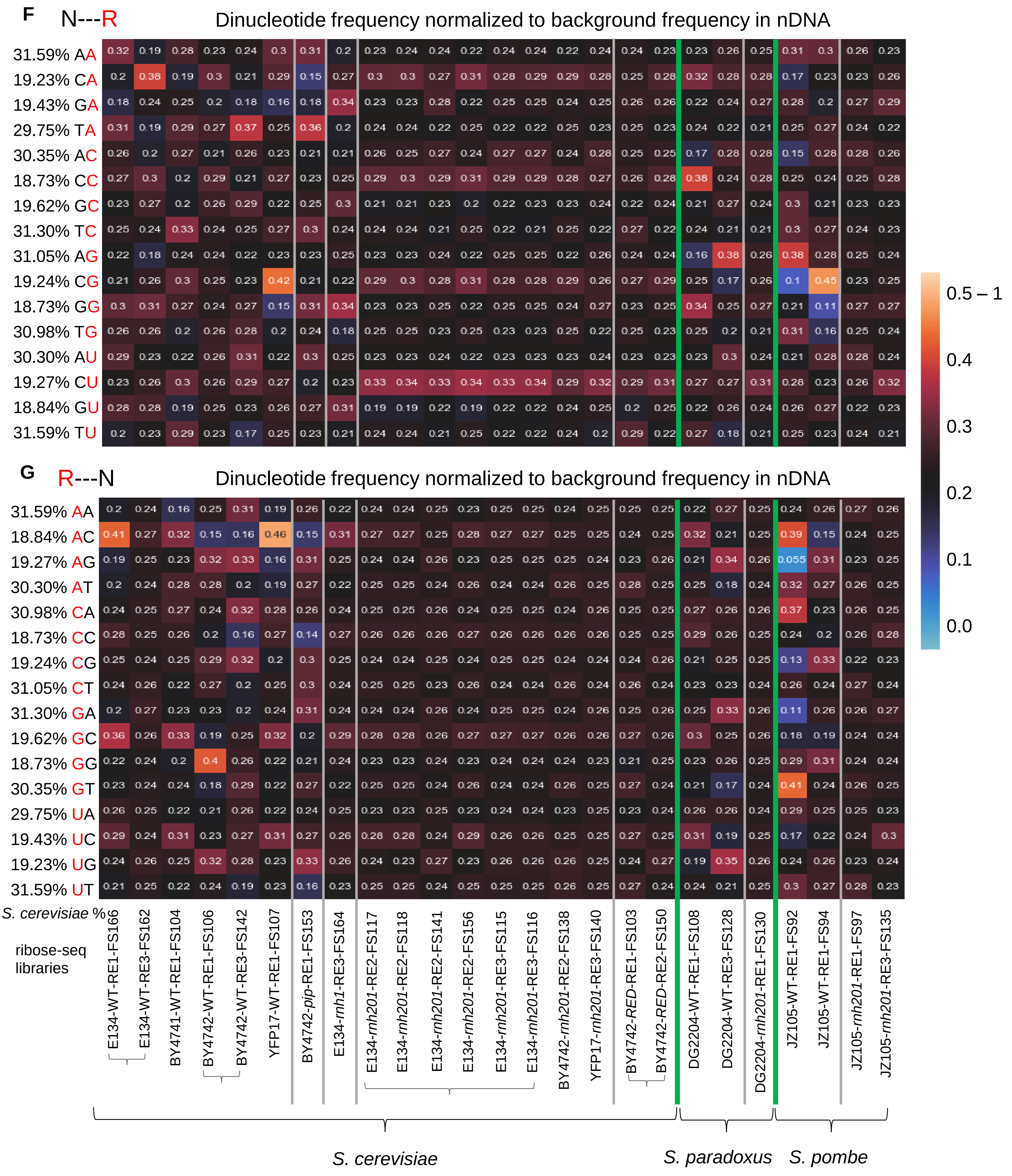
F
N---R
Dinucleotide frequency normalized to background frequency in nDNA
31.59% AA
19.23% CA
19.43% GA
29.75% TA
30.35% AC
18.73% CC
19.62% GC
31.30% TC
31.05% AG
19.24% CG
18.73% GG
30.98% TG
30.30% AU
19.27% CU
18.84% GU
31.59% TU
G
R---N
Dinucleotide frequency normalized to background frequency in nDNA
31.59% AA
18.84% AC
19.27% AG
30.30% AT
30.98% CA
18.73% CC
19.24% CG
31.05% CT
31.30% GA
19.62% GC
18.73% GG
30.35% GT
29.75% UA
19.43% UC
19.23% UG
31.59% UT
S. cerevisiae %
ribose-seq libraries
E134-WT-RE1-FS166
E134-WT-RE3-FS162
BY4741-WT-RE1-FS104
BY4742-WT-RE1-FS106
BY4742-WT-RE3-FS142
YFP17-WT-RE1-FS107
BY4742-pip-RE1-FS153
E134-rnh1-RE3-FS164
E134-rnh201-RE2-FS117
E134-rnh201-RE2-FS118
E134-rnh201-RE2-FS141
E134-rnh201-RE2-FS156
E134-rnh201-RE3-FS115
E134-rnh201-RE3-FS116
BY4742-rnh201-RE2-FS138
YFP17-rnh201-RE3-FS140
BY4742-RED-RE1-FS103
BY4742-RED-RE2-FS150
DG2204-WT-RE1-FS108
DG2204-WT-RE3-FS128
DG2204-rnh201-RE1-FS130
JZ105-WT-RE1-FS92
JZ105-WT-RE1-FS94
JZ105-rnh201-RE1-FS97
JZ105-rnh201-RE3-FS135
S. cerevisiae
S. paradoxus
S. pombe
0.5 – 1
0.4
0.3
0.2
0.1
0.0

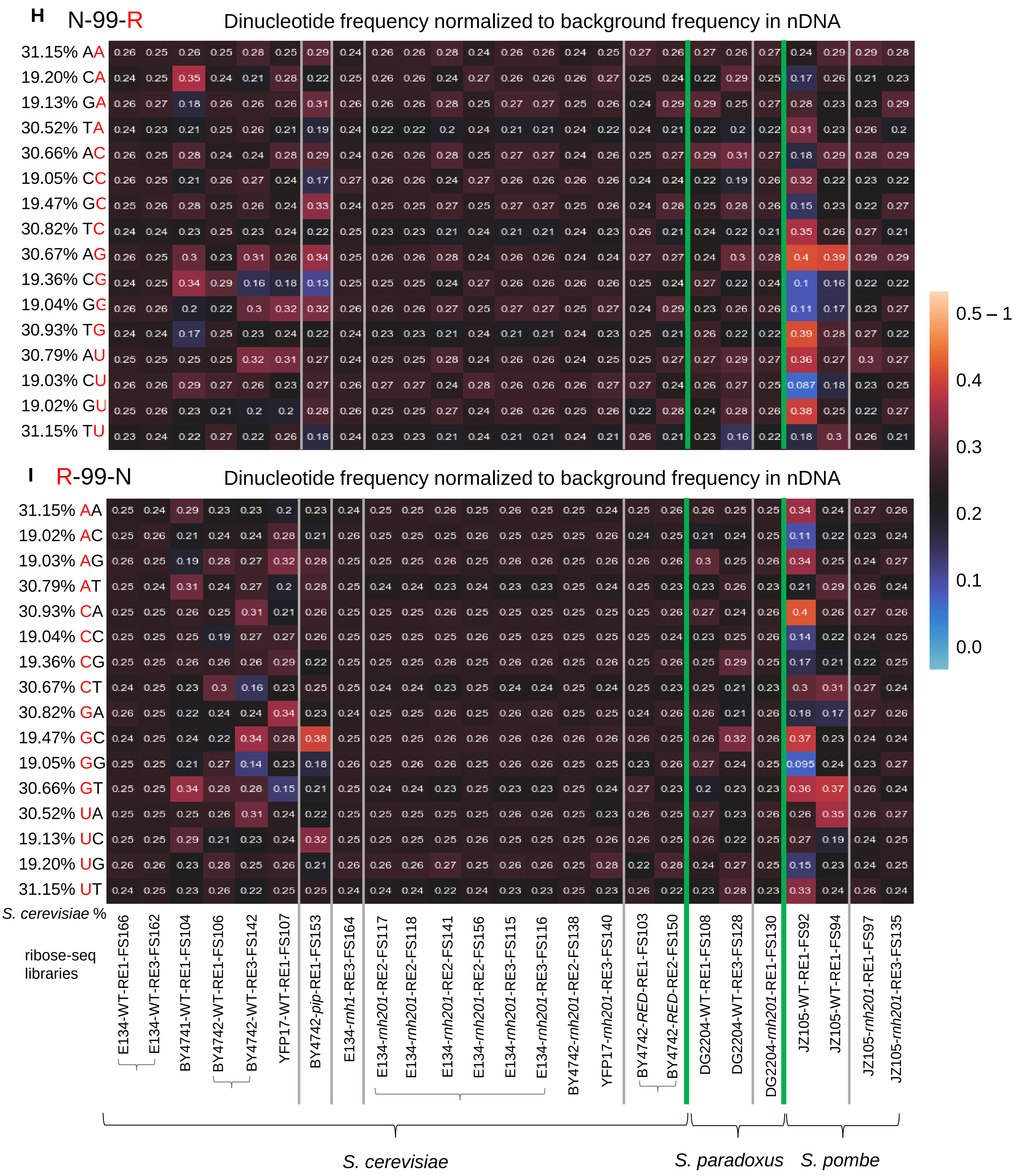
H
N-99-R
Dinucleotide frequency normalized to background frequency in nDNA
31.15% AA
19.20% CA
19.13% GA
30.52% TA
30.66% AC
19.05% CC
19.47% GC
30.82% TC
30.67% AG
19.36% CG
19.04% GG
30.93% TG
30.79% AU
19.03% CU
19.02% GU
31.15% TU
I
R-99-N
Dinucleotide frequency normalized to background frequency in nDNA
31.15% AA
19.02% AC
19.03% AG
30.79% AT
30.93% CA
19.04% CC
19.36% CG
30.67% CT
30.82% GA
19.47% GC
19.05% GG
30.66% GT
30.52% UA
19.13% UC
19.20% UG
31.15% UT
S. cerevisiae %
ribose-seq libraries
E134-WT-RE1-FS166
E134-WT-RE3-FS162
BY4741-WT-RE1-FS104
BY4742-WT-RE1-FS106
BY4742-WT-RE3-FS142
YFP17-WT-RE1-FS107
BY4742-pip-RE1-FS153
E134-rnh1-RE3-FS164
E134-rnh201-RE2-FS117
E134-rnh201-RE2-FS118
E134-rnh201-RE2-FS141
E134-rnh201-RE2-FS156
E134-rnh201-RE3-FS115
E134-rnh201-RE3-FS116
BY4742-rnh201-RE2-FS138
YFP17-rnh201-RE3-FS140
BY4742-RED-RE1-FS103
BY4742-RED-RE2-FS150
DG2204-WT-RE1-FS108
DG2204-WT-RE3-FS128
DG2204-rnh201-RE1-FS130
JZ105-WT-RE1-FS92
JZ105-WT-RE1-FS94
JZ105-rnh201-RE1-FS97
JZ105-rnh201-RE3-FS135
S. cerevisiae
S. paradoxus
S. pombe
0.5 – 1
0.4
0.3
0.2
0.1
0.0

Supplementary Figure 6. dNMP positions having low impact on rNMP occurrence in yeast nDNA. Heatmap analyses with normalized frequency of (**A**) RN, (**B**) N-R, (**C**) R-N, (**D**) N--R, (**E**) R--N, (**F**) N---R, (**G**) R---N, (**H**) N-99-R, and (**i**) R-99-N dinucleotides (rA, rC, rG and rU with the +1, -2, +2, -3, +3, -4, +4, -100 or +100 deoxyribonucleotide with base A, C, G or T) for all the nuclear ribose-seq libraries of this study. The formulas used to calculate these normalized frequencies are shown and explained in Methods. Each column of the heatmaps shows results of a specific ribose-seq library. Each library name is indicated underneath each column of the heatmaps with its corresponding strain name, genotype, and restriction-enzyme (RE) set used. The yeast species of the ribose-seq libraries are also indicated. *S. cerevisiae* libraries derived from the same strains are grouped together by curly brackets. Thick, vertical green lines separate data from the different yeast species. Vertical gray lines separate data obtained from different RNase H genotypes within each species. Each row shows results obtained for a dinucleotide RN (R in red), N-R, R-N, N--R, R--N, N---R, R---N, N-99-R, or R-99-N of fixed rNMP base A, C, G or U for each library. The actual % of dinucleotides of fixed base A, C, G or T for the indicated base combinations that are present in nDNA of *S. cerevisiae* are shown to the left of the heatmaps and are also indicated in Supplementary Data 2A. The corresponding dinucleotide % for nDNA of *S. paradoxus* and *pombe* are indicated in Supplementary Data 2B and 2C, respectively. The observed % of dinucleotides with NMPs with base A, C, G or U were divided by the actual % of each dinucleotide with fixed base A, C, G or T in nDNA of the corresponding species. The bar to the right shows how different frequency values are represented as different colors: black for 0.25; black to yellow for 0.25 to 0.5-1, and black to light blue for 0.25 to 0.

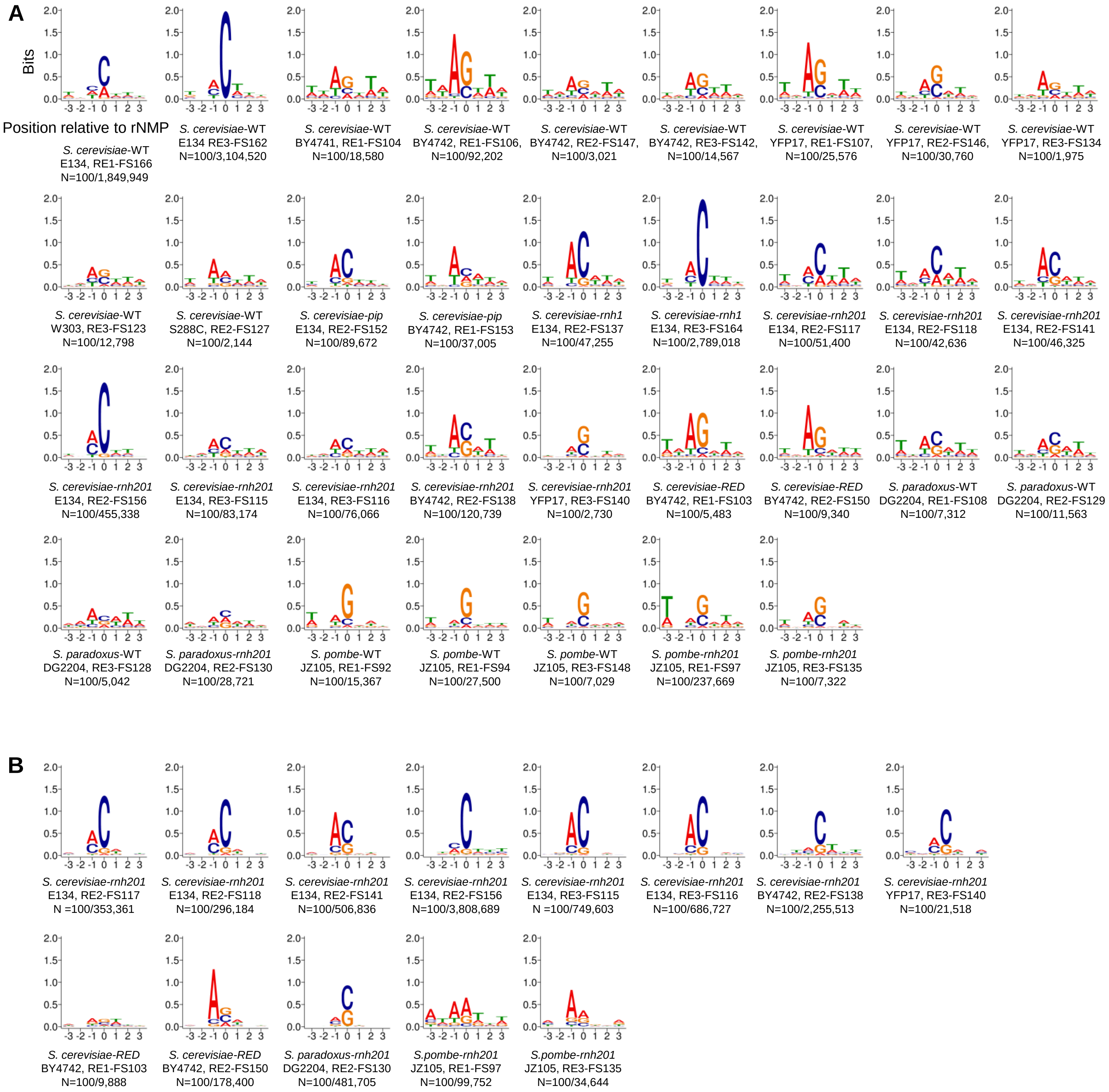

Supplementary Figure 7. Hotspot motifs from mitochondrial and nDNA from top 100 rNMP sites. (**A**) Sequence motif plots for mitochondrial and (**B**) nuclear hotspots (top 100 of rNMP sites). Position 0 on the x-axis represents the site of rNMP incorporation, - and + positions represent upstream and downstream dNMPs, respectively. The y-axis shows the level of sequence conservation, represented in bits. The species, genotype, strain, restriction-enzyme (RE) set, library name, and te number of rNMP sites are included below each plot.

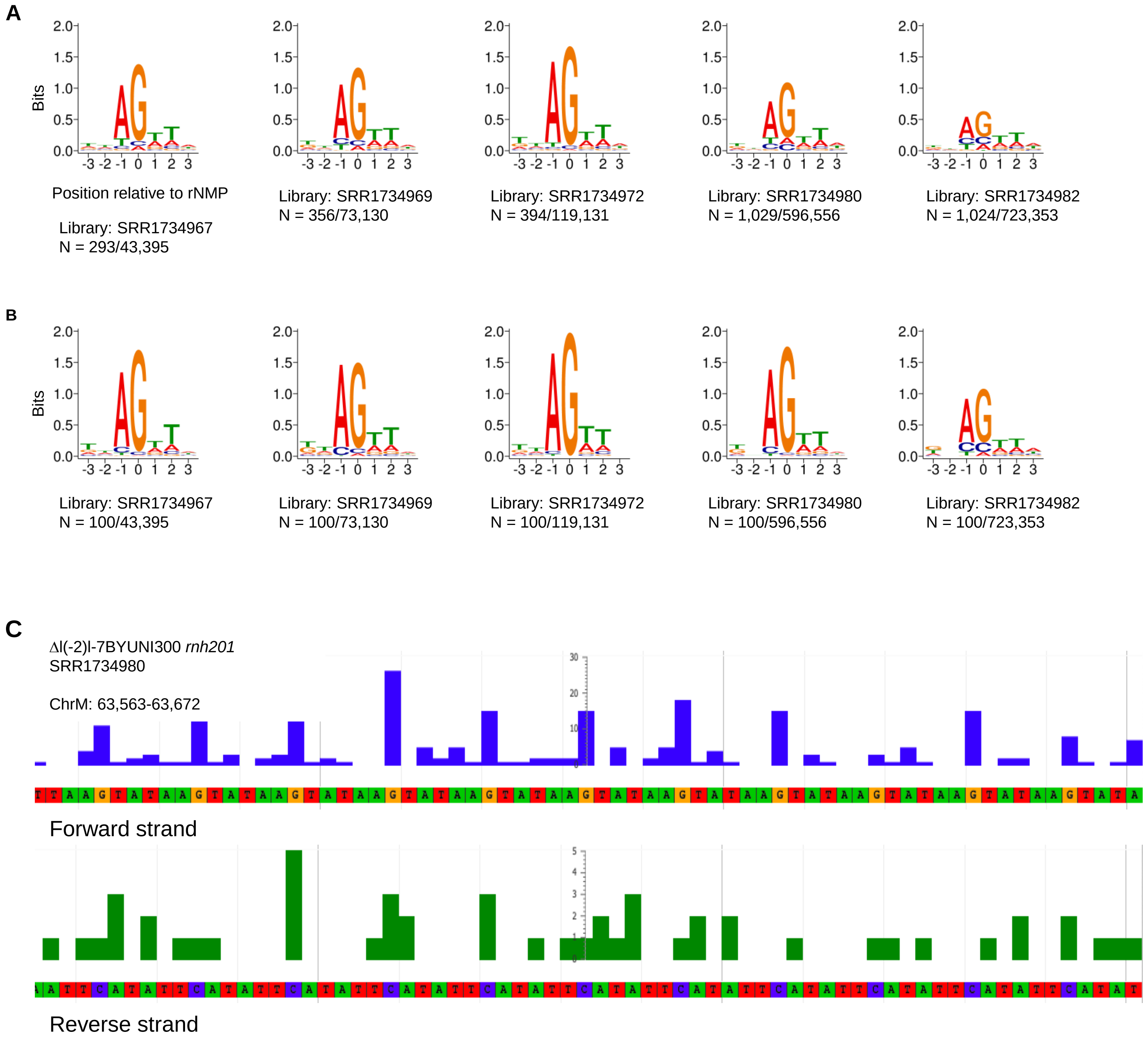

Supplementary Figure 8. Hotspot motifs from mitochondrial and nDNA of emRiboSeq libraries. (**A**) Sequence motif plots for mitochondrial hotspots, top 1% of rNMP sites, and (**B**) top 100 of rNMP sites of emRiboSeq libraries derived from *rnh201*-null *S. cerevisiae* cells. Position 0 on the x-axis represents the site of rNMP incorporation, - and + positions represent upstream and downstream dNMPs, respectively. The y-axis shows the level of sequence conservation, represented in bits. (**C**) Genome browser snapshot showing an rG hotspot within the TAAGTA-repeated sequence on the forward strand and an rC hotspot on the complementary strand in *S. cerevisiae* mtDNA at the locus chrM: 63,563-63,672 for emRiboSeq library SRR1734980 (*rnh201*-null).

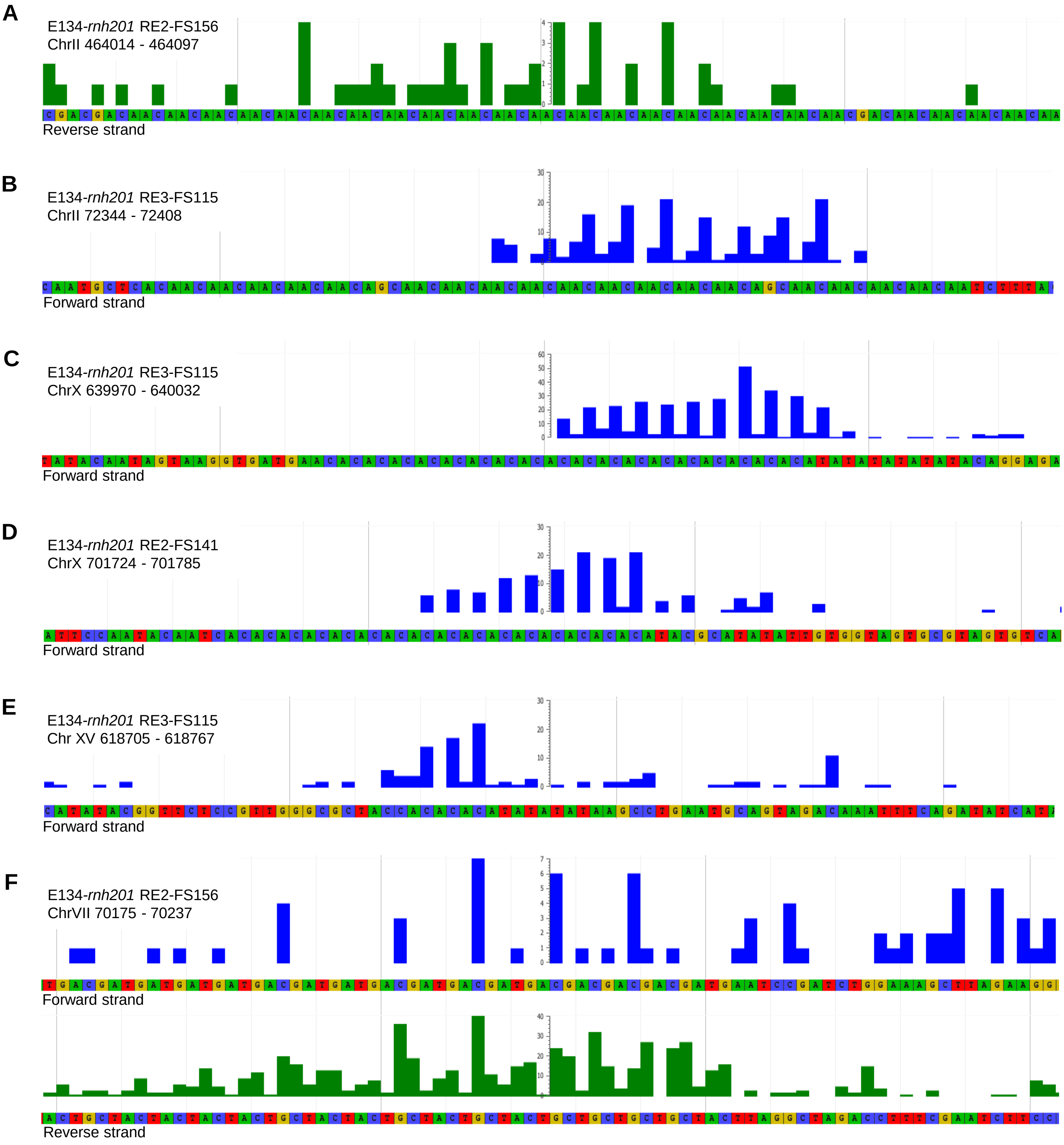

A
E134-*rnh201* RE2-FS156
ChrII 464014 - 464097
Reverse strand
B
E134-*rnh201* RE3-FS115
ChrII 72344 - 72408
Forward strand
C
E134-*rnh201* RE3-FS115
ChrX 639970 - 640032
Forward strand
D
E134-*rnh201* RE2-FS141
ChrX 701724 - 701785
Forward strand
E
E134-*rnh201* RE3-FS115
Chr XV 618705 - 618767
Forward strand
F
E134-*rnh201* RE2-FS156
ChrVII 70175 - 70237
Forward strand
Reverse strand

Supplementary Figure 9. Patterns of rNMPs in tri- and di nucleotide repeat tracts. Genome browser snapshots of *S. cerevisiae* nDNA showing examples of tri- and di-nucleotide repeat tracts with a specific rNMP pattern. **(A)** rC in the repeated-motif AAC at locus chrII:464014..464097 on the reverse strand for library FS156 (E134 *rnh201*Δ RE2), and similarly for FS141 (E134 *rnh201*-null RE2), FS138 (BY4742 *rnh201*-null RE2), FS150 (BY4742 *rnh201-RED* RE2), FS115 and FS116 (E134 *rnh201*-null RE3), FS117 and FS118 (E134 *rnh201*-null RE2). **(B)** Pattern AArC in AAC-repeated sequence at locus chrII:72344..72408 on the forward strand for library FS115 (E134 *rnh201*-null RE3), and similarly for FS116, FS117, FS118, FS138, FS140, FS141, FS156 (E134 *rnh201*-null), and FS150 (BY4742 *rnh201-RED*). **(C)** rC hotspot within the AC-repeated sequence at locus chrX:639970..640032 on the forward strand for library FS115 (E134 *rnh201*Δ RE3), and similarly for FS115 and FS116 (E134 *rnh201*-null RE3), FS117 and FS118 (E134 *rnh201*-null RE2), FS138 (BY4742 *rnh201*-null RE2), FS140 (YFP17 *rnh201*-null RE3), FS141 (E134 *rnh201*-null RE2), FS150 (BY4742 *rnh201-RED* RE2) and FS156 (E134 *rnh201*-null RE2). **(D)** rC hotspot within the AC-repeated sequence at locus chrX:701724..701785 on the forward strand for library FS141 (E134 *rnh201*Δ RE2), and similarly for FS116 (E134 *rnh201*-null RE3), FS117 and FS118 (E134 *rnh201*-null RE2), FS138 (BY4742 *rnh201*-null RE2), FS150 (BY4742 *rnh201-RED* RE2) and FS156 (E134 *rnh201*-null RE2). **(E)** rC hotspot within the AC-repeated sequence at locus chrXV:618732..618740 on the forward strand for library FS115 (E134 *rnh201*Δ RE3), and similarly for FS116 (E134 *rnh201*-null RE3), FS117 and FS118 (E134 *rnh201*-null RE2), FS138 (BY4742 *rnh201*-null RE2), S140 (YFP17 *rnh201*-null RE3), FS141 (E134 *rnh201*-null RE2), FS150 (BY4742 *rnh201-RED* RE2) and FS156 (E134 *rnh201*-null RE2). **(F)** rC hotspot within GArC repeated sequence at locus chrVII:70175..70237 on the forward strand for library FS156 (E134 *rnh201*Δ RE2). The same locus has also rG hotspot on the reverse complement site in TCrG of TGC-repeated sequence, and similarly for FS117, FS118, FS138, FS141 (all *rnh201*-null) and FS150 (BY4742 *rnh201-RED* RE2). This pattern with an rNMP hotspot on both strands in the same site was also seen in mtDNA of *S. cerevisiae* at locus chrM:63583..63651 (see Fig. 7d).

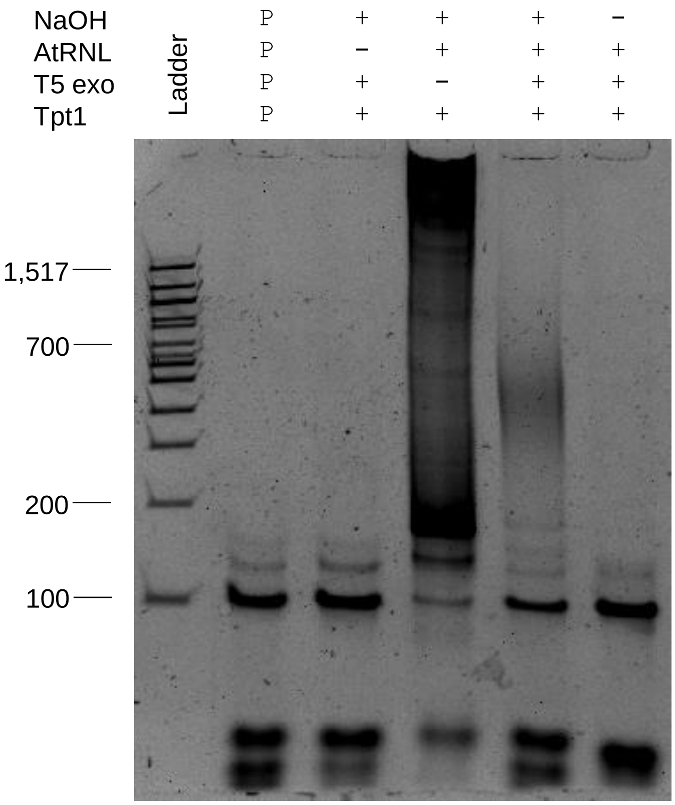

Supplementary Figure 10. Alkali treatment is required for efficient ribose-seq library preparation. PAGE visualization of PCR products associated with a ribose-seq library prepared from *S. cerevisiae rnh201*-null cells using the ribose-seq protocol employed in this study. Lane 1, 100 bp DNA Ladder; lane 2: control with PCR primers only (P); lane 3: control sample alkali+, AtRNL-, T5Exo+ and Tpt1+; lane 4: control sample alkali+, AtRNL+, T5 Exo- and Tpt1+; lane 5: sample of ribose-seq library alkali+, AtRNL+, T5 Exo+ and Tpt1+; lane 6: control sample alkali-, AtRNL+, T5 Exo+ and Tpt1+. PCR cycle numbers for PCR round 1 and PCR round 2 are 6 and 17 cycles, respectively.

Supplementary References

1. Koh, K. D., Balachander, S., Hesselberth, J. R. & Storici, F. Ribose-seq: global mapping of ribonucleotides embedded in genomic DNA. Nat .Meth. 12, 251-257 (2015).
2. Storici, F., Lewis, L. K. & Resnick, M. A. In vivo site-directed mutagenesis using oligonucleotides. Nat. Biotechnol. 19, 773-776 (2001).
3. Brachmann, C. B. et al. Designer deletion strains derived from Saccharomyces cerevisiae S288C: a useful set of strains and plasmids for PCR-mediated gene disruption and other applications. Yeast 14, 115-132 (1998).
4. Keskin, H. et al. Transcript-RNA-templated DNA recombination and repair. Nature 515, 436-439 (2014).
5. Ralser, M. et al. The Saccharomyces cerevisiae W303-K6001 cross-platform genome sequence: insights into ancestry and physiology of a laboratory mutt. Open Biol. 2, 120093 (2012).
6. Mortimer, R. K. & Johnston, J. R. Genealogy of principal strains of the yeast genetic stock center. Genetics 113, 35-43 (1986).
7. Garfinkel, D. J., Nyswaner, K. M., Stefanisko, K. M., Chang, C. & Moore, S. P. Ty1 copy number dynamics in Saccharomyces. Genetics 169, 1845-1857 (2005).
8. Vengrova, S. & Dalgaard, J. Z. RNase-sensitive DNA modification(s) initiates S. pombe mating-type switching. Genes Dev. 18, 794-804 (2004).
