# Supplementary material for: Ribonucleotide incorporation in yeast genomic DNA shows preference for cytosine and guanosine preceded by deoxyadenosine
Source: Nat Commun. 2020 May 15;11:2447. doi: 10.1038/s41467-020-16152-5 (PMC7229183; doi:10.1038/s41467-020-16152-5)
Supplement: Supplementary file 3 — Description of Additional Supplementary Files [file 41467_2020_16152_MOESM3_ESM.pdf]

Description of Additional Supplementary Files

Supplementary Data 1. rNMPs found in mitochondrial ribose-seq libraries.
List of mitochondrial ribose-seq libraries constructed in this study with corresponding data information and indication of yeast species, genotype, strain name, RE set, library name, number of rNMPs, % rA, rC rG and rU with mean and standard deviation for each genotype of a yeast species, barcode and number of cycles in PCR 1 and PCR 2. *When less than three samples were available, the numbers indicate the minimum and maximum range from the mean.

Supplementary Data 2. Background frequency of heatmaps in *S. cerevisiae*, *S. paradoxus* and *S. pombe*.
Frequencies (%) of mononucleotides and dinucleotides of mtDNA and nDNA in (**A**) *S. cerevisiae*, (**B**) *S. paradoxus* and (**C**) *S. pombe* are processed separately. (**A**) According to the sacCer2 genome database, the A+T content in *S. cerevisiae* mtDNA is 83%, with 41.45% deoxyribonucleoside monophosphate (dNMP) with base A, 41.45% T, 8.55% C and 8.55% G; while the A+T content in *S. cerevisiae* nDNA is 62%, with 31.00% deoxyribonucleoside monophosphate (dNMP) with base A, 31.00% T, 19.00% C and 19.00% G. (**B**) According to the ASM207914v1 genome database, the A+T content in *S. paradoxus* mtDNA is 84.7%, with 42.35% A, 42.35% T, 7.65% C and 7.65% G; while the A+T content in *S. paradoxus* nDNA is 61.62%, with 30.81% A, 30.81% T, 19.19% C and 19.19% G. (**C**). According to the PomBase genome database, the A+T content in *S. pombe* mtDNA is 69.91%, with 34.95% A, 34.95% T, 15.045% C and 15.045% G; while the A+T content in *S. pombe* nDNA is 63.95%, with 31.975% A, 31.975% T, 18.025% C and 18.025% G. For the dinucleotide tables, "R" means incorporated ribonucleotide, "N" means deoxyribonucleotide neighbor of base A, C, G or T, and "-" means ignored deoxyribonucleotide in the middle. The raw number of each monoribonucleotide or dinucleotide in the reference genome is counted and the percentage is calculated. For the dinucleotides NR or RN, the position of the R is fixed to either A, C, G or T and the percentage is calculated by dividing the raw number of NR or RN to the sum of the four combinations for each fixed R. E.g. in the NR = AC, the percentage is ACx100/(AC+CC+GC+TC); for RN = AC, the percentage is ACx100/(AA+AC+AG+AT).

Supplementary Data 3. Comparison of frequencies with *P*-values of heatmap data.
Mann-Whitney *U* test for (**A**) mitochondrial and (**B**) nuclear heatmap data. Two-sided Mann-Whitney *U* test is performed in all yeast species for the indicated genotype groups to compare frequencies of mitochondrial or nuclear mononucleotide rNMPs (R), or dinucleotides with an rNMP (NR or RN), within each group. The *P*-value measures the significant level of one rNMP or dinucleotide with an rNMP being greater than another. Mononucleotides were compared to each other. For the dinucleotides NR or RN, the position of the R is fixed to either A, C, G or U, and the comparison is made between the four pairs containing the same fixed R.

Supplementary Data 4. rNMPs found in nuclear ribose-seq libraries.
List of nuclear ribose-seq libraries constructed in this study with corresponding data information and indication of yeast species, genotype, strain name, RE set, library name, number of rNMPs, % rA, rC rG and rU with mean and standard deviation for each genotype of a yeast species, barcode and number of cycles in PCR 1 and PCR 2. *When less than three samples were available, the numbers indicate the minimum and maximum range from the mean.

Supplementary Data 5. Common hotspots sites found in *S. cerevisiae* mitochondrial and nuclear ribose-seq libraries.

Chromosomal coordinates of rNMP sites common to all ribose-seq libraries in the (A) mitochondria of WT S. *cerevisiae* (libraries: FS166, FS162, FS147, FS146, FS142, FS134, FS127, FS123, FS107, FS104, and FS106), (B) mitochondria of *rnh201 S. cerevisiae* (libraries: FS156, FS141, FS140, FS138, FS118, FS117, FS115, FS116), and (C) nucleus of *rnh201 S. cerevisiae* (libraries: FS156, FS141, FS140, FS138, FS118, FS117, FS115, FS116). In addition to the chromosomal coordinates, the total count of rNMPs at a particular coordinate among all libraries, rNMP sequence context of the coordinate (3 nucleotides up/downstream from rNMPs), and genome annotation are provided for each chromosomal coordinate of rNMP incorporation.
