# Supplementary material for: Ribonucleotide incorporation in yeast genomic DNA shows preference for cytosine and guanosine preceded by deoxyadenosine
Source: Nat Commun. 2020 May 15;11:2447. doi: 10.1038/s41467-020-16152-5 (PMC7229183; doi:10.1038/s41467-020-16152-5)
Supplement: Supplementary file 9 — Reporting Summary [file 41467_2020_16152_MOESM9_ESM.pdf]

nature research

Corresponding author(s): NCOMMS-19-18835C

Last updated by author(s): Mar 23, 2020

Reporting Summary

Nature Research wishes to improve the reproducibility of the work that we publish. This form provides structure for consistency and transparency in reporting. For further information on Nature Research policies, see Authors & Referees and the Editorial Policy Checklist.

Statistics

For all statistical analyses, confirm that the following items are present in the figure legend, table legend, main text, or Methods section.

n/a	Confirmed	
☐	☒	The exact sample size (n) for each experimental group/condition, given as a discrete number and unit of measurement
☐	☒	A statement on whether measurements were taken from distinct samples or whether the same sample was measured repeatedly
☐	☒	The statistical test(s) used AND whether they are one- or two-sided<br>*Only common tests should be described solely by name; describe more complex techniques in the Methods section.*
☒	☐	A description of all covariates tested
☒	☐	A description of any assumptions or corrections, such as tests of normality and adjustment for multiple comparisons
☐	☒	A full description of the statistical parameters including central tendency (e.g. means) or other basic estimates (e.g. regression coefficient) AND variation (e.g. standard deviation) or associated estimates of uncertainty (e.g. confidence intervals)
☐	☒	For null hypothesis testing, the test statistic (e.g. F, t, r) with confidence intervals, effect sizes, degrees of freedom and P value noted<br>*Give P values as exact values whenever suitable.*
☒	☐	For Bayesian analysis, information on the choice of priors and Markov chain Monte Carlo settings
☒	☐	For hierarchical and complex designs, identification of the appropriate level for tests and full reporting of outcomes
☒	☐	Estimates of effect sizes (e.g. Cohen's d, Pearson's r), indicating how they were calculated

Our web collection on statistics for biologists contains articles on many of the points above.

Software and code

Policy information about availability of computer code

Data collection	No software was used
Data analysis	Ribose-Map bioinformatics toolkit (https://github.com/agombolay/ribose-map)<br>Custom scripts for background subtraction (https://github.com/xph9876/ArtificialRiboseDetection)<br>Custom scripts for heatmaps (https://github.com/xph9876/RibosePreferenceAnalysis)

For manuscripts utilizing custom algorithms or software that are central to the research but not yet described in published literature, software must be made available to editors/reviewers. We strongly encourage code deposition in a community repository (e.g. GitHub). See the Nature Research guidelines for submitting code & software for further information.

Data

Policy information about availability of data

All manuscripts must include a data availability statement. This statement should provide the following information, where applicable:

- Accession codes, unique identifiers, or web links for publicly available datasets
- A list of figures that have associated raw data
- A description of any restrictions on data availability

The authors declare that the data supporting the findings of this study are available within the paper and its supplementary information files. The ribose-seq datasets generated during the current study are available in NCBI's Sequence Read Archive via BioProject "PRJNA613920 [https://www.ncbi.nlm.nih.gov/bioproject/PRJNA613920]". The emRiboSeq datasets analyzed during the current study are available in NCBI's Gene Expression Omnibus via accession number "GSE64521 [https://www.ncbi.nlm.nih.gov/geo/query/acc.cgi?acc=GSE64521]". The files of Supplementary Data 1,4 and 5 contain raw data.

Field-specific reporting

Please select the one below that is the best fit for your research. If you are not sure, read the appropriate sections before making your selection.

☒ Life sciences ☐ Behavioural & social sciences ☐ Ecological, evolutionary & environmental sciences

For a reference copy of the document with all sections, see nature.com/documents/nr-reporting-summary-flat.pdf

Life sciences study design

All studies must disclose on these points even when the disclosure is negative.

Sample size	In our study, each sample is defined as an individual library of genomic ribonucleotide sites. Each library was prepared and processed independently.
Data exclusions	Four of the ribose-seq libraries had a higher number of background reads that originated from the capture of restriction enzyme ends likely by residual activity of T4 DNA ligase. These background reads were identified computationally and were found to constitute less than 5% of the total reads in the mitochondrial and less than 7.5% nuclear rnh201-null and rnh201-RED libraries. To determine whether the background reads could influence our results and conclusions, we subtracted the background reads from the total reads. When the background reads were <12% of the total reads, the results after background subtraction were found to be the same as those without subtraction. Therefore, for our analyses, we selected only those nuclear libraries for which the background was <12%, so that our results and conclusions are not biased.
Replication	All attempts at replication were successful.
Randomization	Randomization are not relevant for our study as we study the incorporation of ribonucleotides in genomic DNA as a whole rather than specific cells or in specific sequences.
Blinding	Blinding allocation is not relevant to our studies and does not include any clinical data.

Reporting for specific materials, systems and methods

We require information from authors about some types of materials, experimental systems and methods used in many studies. Here, indicate whether each material, system or method listed is relevant to your study. If you are not sure if a list item applies to your research, read the appropriate section before selecting a response.

Materials & experimental systems

n/a	Involved in the study	
☒	☐	Antibodies
☐	☒	Eukaryotic cell lines
☒	☐	Palaeontology
☒	☐	Animals and other organisms
☒	☐	Human research participants
☒	☐	Clinical data

Methods

n/a	Involved in the study	
☒	☐	ChIP-seq
☒	☐	Flow cytometry
☒	☐	MRI-based neuroimaging

Eukaryotic cell lines

Policy information about cell lines

Cell line source(s)	All the yeast strains used in this study are presented in Supplementary Table 1. We used haploid Saccharomyces cerevisiae strains from different backgrounds: E134, BY4741, BY4742, YFP17, W303 and S288C. In addition to S. cerevisiae, we also used Saccharomyces paradoxus (DG2204), and Schizosaccharomyces pombe (JZ105). All these strains are in our laboratory strain collection except for strains BY4741, W303, and S288C which were a gift from the laboratory of Dr. Y. Chernoff at Georgia Institute of Technology, as indicated in the Acknowledgment section.
Authentication	The genotype of the yeast strains were checked by examining that the given genotype of the strains was matching the phenotype of the strains. In addition, we also directly checked specific genotypes that we generate in the study by PCR analysis and sequence analysis.
Mycoplasma contamination	N/A
Commonly misidentified lines (See ICLAC register)	N/A
